# Prevalence of criminal legal involvement among emergency department patients: Insights from the National Survey on Drug Use and Health 2021-2023

**DOI:** 10.1371/journal.pone.0351233

**Published:** 2026-07-08

**Authors:** Vidya Eswaran, Jun-Hong Chen, Utsha G. Khatri, Phillip L. Marotta, Michael G. Vaughn

**Affiliations:** 1 Department of Emergency Medicine, Washington University in St. Louis, St. Louis, Missouri, United States of America; 2 School of Social Work, Saint Louis University, St. Louis, Missouri, United States of America; 3 Department of Emergency Medicine, Icahn School of Medicine at Mount Sinai, New York City, New York, United States of America; 4 Brown School of Social Work and School of Public Health, Washington University. in St. Louis, St. Louis, Missouri, United States of America; University of North Carolina at Chapel Hill, UNITED STATES OF AMERICA

## Abstract

**Introduction:**

Criminal legal involvement (CLI) is a known predictor of short- and long-term adverse health outcomes, and those with CLI often use emergency departments (EDs) to access health care. However, the prevalence of CLI among ED patients is unknown. We sought to assess lifetime and past year CLI among those reporting an ED visit for any reason, for substance use and for mental health, and predictors of ED use among those with CLI to inform efforts to establish ED-based screening and interventions.

**Methods:**

We analyzed data from the 2021–2023 National Survey on Drug Use and Health. Participants reported lifetime CLI, past year CLI and ED visits in the last 12 months for any reason, for substance use and for mental health. We performed descriptive analysis and logistical regression to determine the association between lifetime CLI and ED visits.

**Results:**

Of those reporting an ED visit for any reason, 19% reported lifetime CLI and 3% reported past year CLI. These numbers rose to 44% (lifetime CLI) and 15% (past year CLI) for substance use related ED visits and 29% (lifetime CLI) and 8% (past year CLI) for mental health related ED visits. Those with lifetime CLI had higher odds of an ED visit for any reason (aOR 1.20, 95% CI 1.12–1.29), for substance use (aOR 1.82, 95% CI 1.36,2.43) and for mental health (aOR 1.54, 95%CI 1.19,1.99).

**Conclusions:**

EDs should recognize lifetime CLI as social determinant of health which impacts a significant proportion of their patients.

## Introduction

The United States (US) incarcerates more of its citizens than any other high-income country, with incarceration rates exceeding those of peer nations [1]. In 2022 1.8 million people were incarcerated in US prisons or jails [2]. A history of incarceration has been associated with increased individual risk of chronic physical and mental health conditions even after release, including cardiovascular disease, chronic lung disease, mental health conditions, substance use disorders, and hearing impairment [3–6]. Communities in which incarceration is more common are more likely to see the spread of communicable disease such as sexually transmitted infections [7–9]. Parental incarceration can also have long-term impact on the physical and mental health of their children [10,11].

Prior research has shown that after release from carceral facilities, those who have been incarcerated preferentially access health care in Emergency Departments (EDs). Data from 2008–2011 regrading self-reported past-year incarceration, parole or probation is associated with increased odds of hospitalization and ED utilization and disproportionate hospital expenditures [12]. In linked data analysis, half of all individuals living with HIV who were released from jail in one county had an ED visit within the first year and had five times higher proportion of ED visits for substance use or mental health compared to the general population [13]. Another single-jurisdiction study of older adults released from jail showed similarly high rates of ED use among older adults in the six months post-release [14]. Individuals released from jail are also more likely to visit EDs frequently, and frequent ED use was also associated with frequent jail bookings, as shown in two single-county analyses [15,16]. Long-term, individuals who have experienced incarceration are also less likely to have a usual source of care or receive preventive services compared to those who have not been incarcerated [17].

Incarcerated individuals in the United States suffer a high burden of comorbid conditions, with 40% of state and federal prisoners having a mental health diagnosis [18], and up to 85% either meet criteria for a substance use disorder or were incarcerated for a drug-related offense [19]. It is estimated that 30–45% of those incarcerated in prison have at least one chronic medical condition or disease [20,21], and many report current or prior infectious disease history, such as HIV or Hepatitis C.^20^ Despite the high prevalence of substance use disorders, and opioid use disorder in particular in carceral settings [22], access to evidence-based treatment during incarceration remains limited, including medications for opioid use disorder [23]. Post-release, disparities in access to treatment persist [24–26]. The result of these barriers to care is seen in the high rates of overdose morbidity and mortality in the weeks to months after release [27]. These risks occur in the context of broader social and structural disadvantage: members of racial and ethnic minoritized groups and individuals from lower socioeconomic backgrounds are both more likely to be incarcerated [1,21] and to have poorer health outcomes overall [28,29]. Criminal-legal involvement (CLI) has therefore been described as a social determinant of health [3,30–33].

EDs represent a critical and primary healthcare access point for individuals with current or prior CLI, especially during period of reentry after release [34]. Barriers to timely outpatient care include disruptions in health insurance due to the Medicaid Inmate Exclusion Policy which limits oversight of the medical care offered by carceral facilities and leads to the suspension or termination of Medicaid when a person is incarcerated, though the proposed Medicaid Reentry Act may alleviate this barrier in the future [35,36]. Additional barriers, such as unmet needs for safe housing, food, transportation and employment may supersede engagement with longitudinal medical care, particularly in the immediate post-release period [37,38]. Further, lack of access to medical records from one’s time in custody can lead to interruptions in access to long-term medications upon release and can make it difficult to know what medications and doses of these medications they were receiving [39,40]. Taken all together, these factors may increase reliance on EDs for both acute and ongoing health needs, making EDs a potentially important site for identifying unmet behavioral health and social needs related to CLI.

As EDs increasingly become an important access point to identify and treat substance use disorders [41], and in an era within the US where access to other sources of care, such as inpatient psychiatric facilities and safety-net clinics, may be decreasing [42,43], their role in addressing health disparities related to CLI is gaining attention [16,44]. Between 2018–2019 and 2020–2021, the rate of ED visits for substance use disorder increased from 74.4 to 103.8 per 10,000 population. Even so, inequities in provision of MOUD from EDs have been described [45], and opportunities to identify and treat more patients remain [46]. Understanding which patients are more likely to rely on ED care, and why, may help inform more tailored and equitable ED-based interventions.

Although patients with CLI experience higher risks for adverse health outcomes, it remains unclear whether and how knowledge of CLI adds meaningful information beyond the identification of SUD, psychiatric disease, or other social needs commonly encountered among ED patients. It is known, however that history of CLI can limit opportunities to access housing and food benefits [47,48], and it is likely that those with a history of CLI could benefit from tailored interventions which are trauma-informed and accessible based on their history. While social determinant screening is becoming more common in health care settings [49–51], screening for CLI is rare, and its potential utility in ED care has not been well defined. The existing literature on ED utilization among people with CLI focuses on cohorts with known and recent incarceration, often identified through recent administrative data linkages or uses survey data from over 15 years ago, during which time significant changes in health care policy have been made, such as Medicaid expansion and the Affordable Care Act [12,14–16]. Less is known about the prevalence of lifetime CLI among the broader ED population, or whether individuals with a history of CLI, regardless of how recent, display different patterns of ED utilization. Efforts to characterize CLI among ED populations using natural language model analysis of electronic medical record notes describe low prevalence of CLI but have not been robustly validated [52]. Without such data, designing targeted interventions and understanding the role ED settings can play in addressing substance use and mental health needs in this population remains challenging.

To address this gap in the literature, our study aimed to characterize the prevalence of lifetime CLI among individuals reporting emergency department utilization in the prior year and by examining whether lifetime CLI is associated with differential patterns of ED utilization. Using nationally representative data, we assessed the prevalence of lifetime CLI among individuals reporting ED visits for any reason, as well as ED visits related to substance use and mental health. Furthermore, we examined whether lifetime CLI was associated with an increased odds of ED use after accounting for sociodemographic and health-related factors. While our primary focus was on lifetime CLI, we also evaluated associations between past-year CLI and ED use to provide context regarding the temporal relationship of incarceration and health care use when considering policy solutions.

## Methods

### Study design and setting

We used data from the 2021–2023 National Survey on Drug Use and Health (NSDUH), an annual cross-sectional survey conducted across all 50 US states and the District of Columbia. This is a nationally representative survey using multi-stage probability sampling design to identify civilian, non-institutionalized US residents over the age of 12. Details on the methodology can be found on the Substance Abuse and Mental Health Services Administration website [53]. Given the sampling strategy of NSDUH it is important to contextualize the study understanding that housing insecure and currently incarcerated or otherwise institutionalized individuals are not included, especially given that both those these social risks are associated both with increased risk of incarceration and ED use [16,54,55]. It is thus possible that our results may understate the prevalence of CLI in this population

For the present study, we used data collected from adults 18 years or older in the 2021–2023 surveys – the most recent year for which data were available at the time of data analysis. The data were downloaded on January 21, 2025. We did not have access to information which could identify individual survey participants during or after data collection. Reporting in this manuscript adheres to STROBE guidelines [56].

### Measures

The primary exposure of interest was history of CLI. Lifetime CLI was defined as an answer of “Yes” to the question, “Not counting minor traffic violations, have you ever been arrested and booked for breaking the law? Being “booked” means that you were taken into custody and processed by the police or by someone connected with the courts, even if you were then released”. Those who are booked may subsequently be prosecuted and incarcerated, referred to drug courts or community service, or referred to probation and other community supervision. Past year CLI was defined as an answer of greater than zero to the question “Not counting minor traffic violations, how many times during the past 12 months have you been arrested and booked for breaking a law?” and serves as a marker of new involvement with the criminal legal system.

The primary outcome in this study was the participants’ use of the ED in the past 12 months. This was obtained from the NSDUH question, “During the past 12 months, how many different times have you been treated in an emergency room for any reason?” Answers to this question were reported numerically by respondents. To create a binary variable, we categorized any response greater than 0 visits as “had an ED visit for any reason” and those with zero reported visits as “no ED visits”. Secondary outcomes included frequent (≥ four) ED visits in the past 12 months, any visit to the ED for substance use in the past 12 months and any visit to the ED for mental health in the past 12 months.

Covariates of interest included age, sex, race and ethnicity, insurance status, income, education, geographic location, and report of major depressive episode, serious psychological distress or a substance use disorder. Methodology on the determination of age, sex, and race and ethnicity can be found in the NSDUH codebook [57]. Race and ethnicity were categorized as Non-Hispanic White, Non-Hispanic Black, Non-Hispanic Other, and Hispanic. Non-Hispanic Other included individuals who identified as Non-Hispanic and Native American/Alaskan Native, Native Hawaiian/Other Pacific Islander, Asian, or multiracial. Race and ethnicity were chosen to be included in the model, not to imply any biological justification but as it is known that mass incarceration in the United States disproportionally impacts Black Americans, especially Black men [58], and is likely due, in part, to racism at all levels including structural [59,60]. Understanding their experiences with the health care system will be especially important. Insurance status was aggregated into a binary (yes/no) variable. Individuals responding affirmatively to having Medicare, Medicaid/CHIP, CHAMPUS, private insurance or insurance not otherwise specified were categorized as insured. This dichotomization was done for valid parameter estimation due to the low numbers of positive responses in some of the categories. Income reflected participants’ reported total family income and categorized into four categories, less than $20,000, $20,000-$49,999, $50,000-$74,999 and $75,000 or more. Education refers to the highest level of reported educational attainment. Metropolitan area was determined by the participants’ response of living in a large metro, small metro or non-metro location. For this analysis, metropolitan area was dichotomized to metro or nonmetro. Finally psychiatric comorbidity was ascertained by NSDUH data elements regarding the presence of at least one major depressive episode the past 12 months, serious psychological distress based on the Kesseler psychological distress score and substance use disorder in the past year. Further information on these variables can be found in the NSDUH codebook, searching for the variables ‘AMDEYR, ‘SPDPSTMON’ and ‘UD5ILALANY’ [57].

### Analysis

We first performed descriptive analysis (count, proportion) of demographic characteristics and the exposures of interest (lifetime CLI and past year CLI) in the total population and by ED utilization in the prior 12 months (Table 1). We then performed bivariate analysis using chi-squared tests of categorical variables to assess the relationship between lifetime CLI and past-year ED utilization and demographic characteristics. This was also done for past-year CLI (Table 2).

**Table 1:** Demographic Characteristics applied with complex sample weighting using 2021-2023 merged NSDUH Data.

	All people (n = 139,524)	ED for any reason (n=29,376)	ED for substance use (n=751)	ED for mental health (n = 1,271)
Lifetime CLI (Yes)	17,855 (14.8%)	5,111 (18.6%)	299 (43.7%)	316 (29.2%)
Past Year CLI (Yes)	2,126 (1.3%)	957 (2.6%)	118 (14.8%)	106 (7.5%)
Age				
18 to 29 years old	53,825 (20.0%)	11,778 (19.6%)	342 (22.7%)	742 (34.2%)
30 to 49 years old	53,956 (33.6%)	10,682 (31.7%)	322 (44.7%)	403 (33.9%)
50 or 50 + years old	31,743 (46.4%)	6,916 (48.7%)	87 (32.6%)	126 (31.9%)
Male (yes)	61,926 (48.7%)	11,375 (45.1%)	397 (58.1%)	499 (44.7%)
Race and Ethnicity				
Non-Hispanic White	83,829 (61.7%)	16,583 (61.2%)	411 (60.0%)	690 (61.8%)
Non-Hispanic Black	15,883 (12.1%)	4,613 (15.6%)	104 (12.4%)	173 (14.6%)
Non-Hispanic Others	15,418 (8.9%)	2,843 (6.8%)	96 (7.2%)	163 (5.6%)
Hispanic	24,394 (17.3%)	5,337 (16.4%)	140 (20.4%)	245 (18.0%)
Have health insurance (Yes)	119,282 (87.9%)	25,819 (90.0%)	641 (85.2%)	1,111 (89.7%)
Income				
Less than $20,000	24,052 (15.3%)	7,034 (21.6%)	260 (35.8%)	394 (29.5%)
$20,000 - $49,999	37,900 (27.0%)	9,319 (31.2%)	236 (30.6%)	431 (30.6%)
$50,000 - $74,999	20,701 (15.4%)	4,152 (14.7%)	86 (9.9%)	161 (14.9%)
$75,000 or More	56,871 (42.3%)	8,871 (32.5%)	169 (23.7%)	285 (25.0%)
College or above (Yes)	48,691 (32.7%)	6,748 (23.4%)	93 (14.3%)	168 (17.5%)
Metropolitan area (Yes)	117,090 (86.0%)	23,751 (83.7%)	624 (85.0%)	1,041 (85.3%)
Major depressive episodes (Yes)	15,340 (8.5%)	4,820 (12.5%)	258 (30.6%)	585 (46.6%)
Serious psychological distress (Yes)	14,973 (7.7%)	4,963 (12.2%)	320 (33.2%)	580 (41.6%)
Substance use disorder (Yes)	29,020 (17.9%)	7,997 (23.0%)	598 (77.8%)	678 (52.1%)

Percentage applied with complex sample weighting is shown in table

ED: Emergency Department; CLI: Criminal Legal Involvement

**Table 2:** Demographic Characteristics by Lifetime and Past Year Criminal Legal Involvement using 2021-2023 merged NSDUH Data (applied with complex sample weighting).

	Lifetime CLI (yes) n = 17,855	Lifetime CLI (no) n = 121,669	Chi-square result	Past Year CLI (yes) n = 2,126	Past Year CLI (no) n = 137,398	Chi-square result
ED visit for any reason (yes)	5,111 (28.9%)	24,054 (22.1%)	p < 0.001	957 (48.0%)	28,174 (22.7%)	p < 0.001
ED visit for substance use (yes)	299 (2.1%)	424 (0.5%)	p < 0.001	118 (8.2%)	602 (0.6%)	p < 0.001
ED visit for mental health (yes)	316 (2.1%)	928 (0.9%)	p < 0.001	106 (6.5%)	1,137 1.0%)	p < 0.001
Age			p < 0.001			p < 0.001
*18 to 29 years old*	4,051 (11.3%)	48,747 (21.6%)		1,088 (29.9%)	51,582 (20.0%)	
*30 to 49 years old*	9,646 (44.2%)	43,239 (31.7%)		875 (44.8%)	51,955 (33.4%)	
*50 or 50 + years old*	4,158 (44.5%)	27,085 (46.7%)		163 (25.3%)	31,049 (46.6%)	
Male (yes)	11,078 (70.3%)	49,602 (44.9%)	p < 0.001	1,304 (70.5%)	59,286 (48.3%)	p < 0.001
Race			p < 0.001			p < 0.001
*Non-Hispanic White*	11,265 (65.4%)	71,264 (61.3%)		1,006 (53.8%)	81,489 (62.0%)	
*Non-Hispanic Black*	2,430 (13.9%)	13,017 (11.7%)		456 (21.1%)	14,961 (11.9%)	
*Non-Hispanic Others*	1,694 (5.8%)	13,429 (9.4%)		260 (6.1%)	14,850 (8.9%)	
*Hispanic*	2,466 (14.9%)	21,361 (17.6%)		404 (19.0%)	23,386 (17.2%)	
Have health insurance (Yes)	15,028 (85.9%)	103,669 (89.3%)	p < 0.001	1,618 (74.9%)	117,003 (89.0%)	p < 0.001
Income			p < 0.001			p < 0.001
*Less than $20,000*	3,993 (20.8%)	19,477 (14.2%)		800 (36.0%)	22,622 (14.9%)	
*$20,000 - $49,999*	5,516 (30.4%)	31,569 (26.4%)		727 (35.3%)	36,321 (26.9%)	
*$50,000 - $74,999*	2,626 (15.1%)	17,691 (15.4%)		215 (11.9%)	20,083 (15.4%)	
*$75,000 or More*	5,720 (33.7%)	50,334 (44.0%)		384 (16.8%)	55,660 (42.8%)	
College or above (Yes)	3,896 (20.2%)	44,183 (35.1%)	p < 0.001	136 (7.2%)	47,937 (33.3%)	p < 0.001
Metropolitan area (Yes)	14,300 (83.6%)	100,586 (86.4%)	p < 0.001	1,614 (78.5%)	113,179 (86.1%)	p < 0.001
Major depressive episodes (Yes)	2,482 (11.8%)	12,794 (8.0%)	p < 0.001	408 (16.4%)	14,861 (8.4%)	p < 0.001
Serious psychological distress (Yes)	2,512 (11.4%)	12,091 (7.0%)	p < 0.001	559 (23.3%)	14,028 (7.5%)	p < 0.001
Substance use disorder (Yes)	7,270 (35.9%)	20,933 (14.5%)	p < 0.001	1,316 (58.7%)	26,839 (17.2%)	p < 0.001

Percentage applied with complex sample weighting is shown in table, ED: Emergency Department, CLI: Criminal Legal Involvement

To address potential false discovery, a Bonferroni correction was applied, and statistical significance was assessed using the adjusted significance threshold. The results remained unchanged.

Demographic characteristics included were age, gender, race and ethnicity, health insurance, income, educational attainments, living at metro area or not, major depressive episodes, serious psychological distress, and substance use disorder. We chose Non-Hispanic White to serve as the reference group as these individuals were the most represented in the sample. We applied complex survey weighting as recommended by the NSDUH Codebook in all analyses [53].

We then employed binary logistic regression models to examine the association between CLI and three ED visit outcomes, ED visit in the past 12 months for any reason (Table 3), ED visit in the past 12 months for substance use and ED visit in the past 12 months for mental health (S1 Table). Using Little’s Test [61], we confirmed that the missing data were missing at random, suggesting that missing data was not associated with demographic characteristics.

**Table 3:** Odds of ED Visit for Any Reason using 2021-2023 merged NSDUH Data (N = 139,524) (applied with complex sample weighting).

	Lifetime CLI			Past Year CLI		
	Odds ratio	*p*-value	95% C.I.	Odds ratio	*p*-value	95% C.I.
**Main Predictor**						
Lifetime CLI (Yes)	**1.20**	**<0.001**	**[1.12, 1.29]**	**2.21**	**<0.001**	**[1.82, 2.69]**
**Demographic Covariates**						
Age						
18 to 29 years old (reference)						
30 to 49 years old	**1.16**	**<0.001**	**[1.09, 1.24]**	**1.19**	**<0.001**	**[1.11, 1.27]**
50 or 50 + years old	**1.32**	**<0.001**	**[1.23, 1.42]**	**1.35**	**<0.001**	**[1.25, 1.45]**
Male (yes)	**0.84**	**<0.001**	**[0.80, 0.89]**	**0.85**	**<0.001**	**[0.81, 0.90]**
Race and Ethnicity						
Non-Hispanic White (reference)						
Non-Hispanic Black	**1.33**	**<0.001**	**[1.23, 1.44]**	**1.32**	**<0.001**	**[1.22, 1.42]**
Non-Hispanic Others	**0.78**	**<0.001**	**[0.70, 0.87]**	**0.78**	**<0.001**	**[0.70, 0.86]**
Hispanic	0.94	0.163	[0.86, 1.03]	0.93	0.106	[0.86, 1.02]
Have one or more health insurance (Yes)	**1.31**	**<0.001**	**[1.20, 1.44]**	**1.33**	**<0.001**	**[1.21, 1.46]**
Income						
Less than $20,000 (reference)						
$20,000 - $49,999	**0.78**	**<0.001**	**[0.72, 0.85]**	**0.79**	**<0.001**	**[0.73, 0.86]**
$50,000 - $74,999	**0.64**	**<0.001**	**[0.58, 0.71]**	**0.65**	**<0.001**	**[0.59, 0.71]**
$75,000 or More	**0.56**	**<0.001**	**[0.51, 0.61]**	**0.56**	**<0.001**	**[0.51, 0.61]**
College or above (Yes)	**0.67**	**<0.001**	**[0.63, 0.71]**	**0.66**	**<0.001**	**[0.63, 0.71]**
Live in metropolitan area (Yes)	**0.90**	**0.007**	**[0.83, 0.97]**	**0.90**	**0.010**	**[0.83, 0.97]**
Major depressive episodes (Yes)	**1.36**	**<0.001**	**[1.25, 1.48]**	**1.37**	**<0.001**	**[1.26, 1.49]**
Serious psychological distress (Yes)	**1.55**	**<0.001**	**[1.42, 1.68]**	**1.53**	**<0.001**	**[1.41, 1.67]**
Substance use disorder (Yes)	**1.39**	**<0.001**	**[1.30, 1.48]**	**1.39**	**<0.001**	**[1.31, 1.47]**

ED: Emergency Department, CLI: Criminal Legal Involvement

For causal inference that accounts for potential selection bias in CLI, we applied propensity score weighting [62,63] to ensure the treatment group (i.e., people with CLI) and the control group (i.e., people without CLI) had similar demographic characteristics and similar baseline psychological distress (i.e., no significant difference between the control and exposed groups).

To assess primary and secondary outcomes by CLI, we employed average treatment effects using propensity score weight. To further reduce estimation bias, we isolated the effects of CLI from demographic features using doubly robust estimation approach [64,65]. Doubly robust estimation is a statistical method used in causal inference to estimate the effect of an intervention or treatment on an outcome variable. While propensity score weight aims to balance the covariates between different subgroups, the robust estimation approach helps differentiate out the covariate effects [66].

We combined two different estimation techniques to improve the accuracy of the results. The first component involved fitting a model to predict the outcome variable based on covariates, which helps to adjust for potential confounding factors. The second component involved fitting a model to predict the treatment assignment based on covariates. Simultaneous application of these approaches ensured the treatment group and the control group shared similar demographic features and also differentiated the treatment effects related to these demographic features [64,65].

Following Guo and Fraser [67] to create a propensity score weight, a logistic regression model was first run to predict the probability of CLI, ***p***. Demographic characteristics (i.e., age, gender, race and ethnicity, health insurance, income, educational attainments, living at metro area or not, major depressive episodes, serious psychological distress, and substance use disorder) were included as independent variables to predict probability ***p***, and were chosen based on prior literature regarding factors associated with risk of incarceration. Next, we utilized the predicted probability ***p*** to create propensity score weights in the following way: For ATT weights, the propensity score weight for people with CLI was ***1***, and for people without CLI was ***p/(1-p)***. These propensity score weighting values were estimated based on the following assumptions: stable unit treatment value assumption (SUTVA) and positivity. SUTVA states that the potential outcome for one individual is not affected by the treatment assignment of another individual. Positivity states that every individual has a nonzero probability of receiving either treatment. (S2 Table).

To assess the association between demographic characteristics and primary and secondary outcomes among people with CLI, we applied binary logistic regression models (Table 4, S3 Table). We used Stata 18.0 Standard Version for all analyses.

**Table 4:** Subgroup Analysis of Criminal Legal-Involved Individuals on ED Visit for Any Reason using 2021-2023 NSDUH Data (applied with complex sample weighting).

Demographic Covariates	People with Lifetime CLI (n = 17,855)		
	Odds ratio	*p*-value	95% C.I.
Age			
18 to 29 years old (reference)	**–**	**–**	**–**
30 to 49 years old	0.94	0.376	[0.82, 1.08]
50 or 50 + years old	0.95	0.561	[0.81, 1.13]
Male (yes)	**0.75**	**<0.001**	**[0.66, 0.85]**
Race and Ethnicity			
Non-Hispanic White (reference)	**–**	**–**	**–**
Non-Hispanic Black	**1.22**	**0.031**	**[1.02, 1.47]**
Non-Hispanic Others	0.94	0.625	[0.75, 1.19]
Hispanic	**0.74**	**0.007**	**[0.60, 0.92]**
Have one or more health insurance (Yes)	1.15	0.187	[0.93, 1.41]
Income			
Less than $20,000 (reference)	**–**	**–**	**–**
$20,000 - $49,999	**0.72**	**<0.001**	**[0.61, 0.85]**
$50,000 - $74,999	**0.54**	**<0.001**	**[0.43, 0.67]**
$75,000 or More	**0.54**	**<0.001**	**[0.47, 0.63]**
College or above (Yes)	**0.62**	**<0.001**	**[0.53, 0.73]**
Live in metropolitan area (Yes)	**0.81**	**0.025**	**[0.67, 0.97]**
Major depressive episodes (Yes)	**1.64**	**<0.001**	**[1.34, 2.01]**
Serious psychological distress (Yes)	1.12	0.240	[0.92, 1.36]
Substance use disorder (Yes)	**1.36**	**<0.001**	**[1.19, 1.55]**

ED: Emergency Department, CLI: Criminal Legal Involvement

## Results

### Characteristics of study subjects

In total 139,524 individuals were included in the study. Of these 17,855 (15%) reported lifetime CLI and 2,126 (1%) reported past year CLI. Most participants identified as Non-Hispanic White (83,829, 62%) and the majority (119,282, 88%) were insured. One third of participants, 48,691 (33%), reported college education or above, and the plurality reported a total family income of $75,000 or more (56,871, 42%). A minority of participants reported a history of major depressive episodes (15,340, 8%) or serious psychological distress (14,973, 8%) and nearly one fifth of respondents (29,020, 18%) reported a substance use disorder. Of the 139,524 respondents, 29,376 reported an ED visit in the 12 months prior for any reason. Fewer reported ED visits in the past 12 months for substance use (n = 751) or mental health (n = 1,271). (Table 1)

### Main Results

For those with past year CLI, 957 (48%) reported a past year ED visit for any reason, 118 (8%) for substance use and 106 (6%) for mental health. Among those with lifetime CLI, the proportion with ED visits was significantly higher than those without CLI. Of the 17,855 with reported lifetime CLI, 5,11 (29%) reported an ED visit for any reason, 299 (2%) an ED visit for substance use, and 316 (2%) an ED visit for mental health (compared to 22%, 0.5% and 0.9% respectively for those without any lifetime CLI p < 0.001 for all comparisons). (Table 2)

Significant differences in all measured demographic characteristics were noted between those with and without lifetime CLI. Similarly significant results were found among those with past year CLI. (Table 2)

### Regression analysis

After adjusting for age, sex, race and ethnicity, income, education, geographic location and behavioral health comorbidity, in logistic regression model with complex sample weighting, those with a history of lifetime CLI had significantly higher odds of an ED visit for any reason (aOR 1.20, 95% CI 1.12–1.29) than those without lifetime CLI. In this model men had lower odds than women to have an ED visit (aOR 0.84, 95%CI 0.80–0.89) and non-Hispanic Black respondents had higher odds of an ED visit than non-Hispanic White respondents (aOR 1.33, 95% CI 1.23–1.44). Having higher income and educational attainment were associated with lower odds of ED visits, as was living in a metropolitan area, while having insurance was associated with higher odds of an ED visit. Finally, having a behavioral health comorbidity led to higher odds of an ED visit for any reason. (Table 3)

In a similar model, those with past year CLI also had significantly higher odds of an ED visit for any reason (aOR 2.21, 95% CI 1.82–2.69) than those without past year CLI. Similar estimates were seen in this model for other demographic covariates as noted for lifetime CLI. (Table 3)

Those with lifetime CLI also had higher odds of a substance use related ED visit (aOR 1.82, 95% CI 1.36, 2.43) and a mental health related ED visit (aOR 1.54, 95%CI 1.19, 1.99) compared to those without lifetime CLI. (S1 Table). When using ATT propensity score weighting, similar results were obtained. (S2 Table)

#### Subgroup analysis.

In subgroup analysis among individuals with lifetime CLI we found that men continued to have lower odds of an ED visit (aOR 0.75, 95%CI 0.66–0.85) and non-Hispanic Black individuals with higher odds (aOR 1.22, 95%CI 1.02–1.47) while Hispanic individuals had lower odds (aOR 0.74, 95%CI 0.60–0.92). Sex, race, income, education, geography and presence of major depressive episodes or substance use disorders were significantly associated with ED use for any reason while age and insurance status was not. (Table 4) However, presence of health insurance was positively associated with ED visit for substance use (aOR 2.42, 95% CI 1.35, 4.34) and mental health (aOR 2.85, 95% CI 1.25, 2.49) while sex, race, college education and geography were not (S3 Table).

## Discussion

The aim of the present study was to leverage a large and well characterized data source to examine the prevalence of lifetime and past year CLI among those using the ED. Our data show that while only 2% of those with an ED visit in the past year for any reason report past year CLI, nearly one in five individuals who had an ED visit reported a history of lifetime CLI. This number rose to over 40% of individuals with a substance related ED visit and nearly 30% of those with a mental health related ED visit. Further, we found that lifetime CLI was associated with higher odds of an ED visit for any reason, as well as ED visits of substance use and mental health related reasons.

This high prevalence of lifetime CLI among patients seeking care for substance-related reasons matches data from community surveys of people who use drugs [68] and recent studies on those under community supervision for CLI [69]. While prior data have shown high rates of ED use in the immediate post-release period, even up to one year [34,70,71], our findings suggest that the association between CLI and ED exists beyond this known high-risk period. A study using a large language model to identify history of incarceration among ED patients found the prevalence to be much lower (<1%), indicating that documentation of incarceration history in EHRs may not be robust, and underscores the potential importance of screening for this social determinant of health [72].

Analyzing lifetime CLI offers critical insight into the long-term effects of systemic criminalization and its potential impact on health behaviors, trust in institutions, and access to care. More specifically, someone with a history of incarceration or arrest—even decades ago—may still carry the psychological, social, and economic consequences of that experience, including medical mistrust or ongoing stigma. Therefore, assessing lifetime CLI can help ED providers and researchers better understand and address persistent disparities in care that a one-year snapshot might miss.

Individuals with a history of CLI face numerous barriers to health care access post-release. In many states a person’s Medicaid is suspended or, worse, terminated while incarcerated and may require the individual to re-apply at release for resumption of benefits [73,74]. Individuals with a history of CLI are less likely to have a primary care home or to receive recommended screenings (such as mammograms or colonoscopies) [17,75]. Compounded with barriers related to housing and financial insecurity commonly faced post-release from incarceration, patients may turn to EDs, America’s safety net, for their health-related needs. In our data those with CLI were slightly, but significantly, less likely to report health insurance than those without CLI, and presence of insurance was in fact associated with higher odds of ED utilization. Other data have shown that while policy efforts related to Medicaid expansion lead to increased insurance coverage among those with past-year CLI, there was no change in the receipt of treatment for substance use disorders or mental health conditions [76]. This finding warrants further exploration to understand whether health insurance coverage translates into actual health care access and use of preventive or longitudinal care, especially the care needed for managing substance use and co-occurring condition, and if not, to understand what other barriers these populations face to accessing outpatient care.

Our findings underscore the importance of considering and addressing CLI as a critical contextual factor in the treatment of patients with substance use and mental health disorders. While individuals with a history of CLI have been shown to be more likely to receive substance use disorder treatment than those without CLI, those that receive treatment still represent a minority of all those that could benefit [77].

There is growing understanding of the importance of competency in the provision of trauma informed care among ED clinicians [78]. Given the significant trauma experienced by many while incarcerated [79], that mistrust of health care systems and providers persist after release from carceral facilities, and that many people who were formally incarcerated report bias from healthcare providers [80,81], knowing that 20% of ED patients have experienced incarceration can be helpful in motivating clinicians to be prepared to deliver trauma-informed care. Practically, this could include lectures to medical students and trainees about mass incarceration and the short- and long-term health impacts, including personal or familial incarceration in lectures related to social determinants of health, inviting individuals with lived experience of incarceration to share their experience in lectures or small groups, and frequent training and skills building in best practices related to the provision of trauma informed care.

Incarceration has been associated with increased risk of adverse medical and behavioral health outcomes and premature mortality [7,82–84]. It is important that ED clinicians are aware of these risks and consider them when developing treatment plans and prioritizing patient referrals for outpatient care. Beyond, individual level care, awareness of high prevalence of CLI among ED patients is important for health systems when considering interventions and resource development. EDs have long been centers of implementation for public health screening and intervention [85–87]. Multiple ED-based programs exist to address basic needs such as food and housing insecurity, for example [88–90]. Universal screening of ED patients for HIV have improved rates of diagnoses and linkage to care [91]. Given proposed and recent changes to Medicaid policy, more individuals may lose access to Medicaid and thus rely on EDs for care beyond their acute, emergent needs [92]. Awareness of CLI among a large portion of ED patients should inspire careful consideration by ED and hospital leadership to consider how ED staff and health care systems can best support this population. ED-based social workers and case managers may be able to connect patients with re-entry focused resources within their community to provide continued support beyond the hospital walls. Medical-Legal partnerships [93,94] could be developed to bring comprehensive social services to patients being seen in the ED. Collaboration between addiction medicine, mental health clinics and EDs could include warm handoffs between clinical sites, and assistance with implementation of protocols to facilitate the provision of medications for substance use disorders in the ED to patients who may otherwise have limited access to outpatient healthcare, especially given known disparities in access to standard of care treatments among those with CLI. The ED could be a meaningful point of intervention and partner to prevent adverse health outcomes, and potentially recidivism and re-incarceration as well.

EDs routinely screen patients for a variety of conditions including domestic violence, suicidality, substance use disorders, travel history and risk of falls [95]. Before wholescale screening for CLI is implemented in EDs, however, it will be important to understand the acceptability of ED-based screening among CLI populations and to thoughtfully consider how and when such screening would take place to minimize undue burden on ED staff. Further, patients with a history of CLI have reported experiencing biased care from health care providers [80,96]. Data from ED nurses, advanced practice providers and physicians show the belief that incarcerated patients receive differential care [97,98]. Data from Internal Medicine healthcare providers demonstrate that clinicians are unsure on how to ask about patients’ CLI [99]. We encourage involvement of individuals with CLI history and community organizations whose work center these populations when considering how to screen patients and how to act on positive screens to ensure screening does not increase actual or perceived discrimination by health care providers. Partnering with local correctional health authorities to facilitate data exchange could also help clinicians in and out of carceral settings understand the health needs of the patients they share.

Despite the uniqueness of our findings, the present study should be interpreted in light of several limitations. First, the data included in our analyses were based on self-report. Self-report data, by their nature, rely on the recall of study participants and are vulnerable to over- and under-reporting of behaviors. Other studies, however, have demonstrated consistency between official records and self-report of justice-involved individuals [100–102]. NSDUH surveys are administered on computer in a private setting which has been shown to minimize under-reporting. Second, NSDUH data are the result of a series of cross-sectional surveys. Due to issues of temporal ordering of study variables, any causal claims cannot be advanced. Third, there may be several variables omitted from the NSDUH that could serve to confound our results. As such, triangulating from multiple data sources in the future is necessary to achieve robustness in the patterns we have identified. Fourth, the use of ‘arrest and booking’ to define CLI, especially in the past-year, limits CLI to new episodes of criminal legal involvement and fails to capture those under community supervision who, while still ‘involved’ with the system, may not have been arrested or booked during the prior twelve months, suggesting prevalence of past year CLI may in truth be higher than our results suggest. The use of lifetime CLI is intended to more broadly include this population. Finally, the NSDUH sampling strategy excludes those in facilities such as jails and prisons as well as those experiencing homelessness but not living in shelters, potentially limiting the generalizability of the results as these populations are more likely to use ED services [16]. Given this is a nationwide sample, regional differences in prevalence likely exist between urban, suburban and rural settings and by local/state jurisdiction. Further work is needed to examine differences between settings such that health care organizations, carceral facilities and policymakers can identify where best to target resources.

In summary, our data suggest a non-insignificant proportion of ED patients may have a history of lifetime CLI, and that CLI history is associated with increased odds of ED use. The ED is uniquely positioned to address both the health and social needs of this highly vulnerable population and should be leveraged as a critical site for intervention and collaboration between EDs and outpatient care resources. Further work which is community based and patient-centered will be needed to formulate and implement systems of screening and intervention to best support these patients.

## Supporting information

S1 Table Odds of ED visit for substance use and mental health in the past 12 months (applied with complex sample weighting). Table 1a. Odds of Substance Use ED Visit, Table 2a. Odds of Mental Health ED Visit.
(DOCX)

S2 Table Odds of ED visit for any reason, substance use and mental health in past 12 months (ATT Model). Table 2a: Odds of ED Visit for Any Reason in Past 12 Months (ATT model), Table 2b: Odds of ED Visit for Substance Use in Past 12 Months (ATT model), Table 2c. Odds of ED Visit for Mental Health in Past 12 Months (ATT model).
(DOCX)

S3 Table Subgroup analysis of lifetime criminal legal-involved individuals on ED visits for substance use and mental health in past 12 months. Table 3a. Subgroup Analysis of Lifetime Criminal Legal-Involved Individuals on ED Visit for Substance Use in Past 12 Months. Table 3b. Subgroup Analysis of Lifetime Criminal Legal-Involved Individuals on ED Visit Mental Health in Past 12 Months.
(DOCX)
