# Supplementary material for: Prevalence of criminal legal involvement among emergency department patients: Insights from the National Survey on Drug Use and Health 2021-2023
Source: PLoS One. 2026 Jul 8;21(7):e0351233. doi: 10.1371/journal.pone.0351233 (PMC13345442; doi:10.1371/journal.pone.0351233)
Supplement: S1 Table — Table 1a. Odds of Substance Use ED Visit, Table 2a. Odds of Mental Health ED Visit. (DOCX) [file pone.0351233.s001.docx]

**S1 Table.** **Odds of ED Visit for Substance Use and Mental Health in the Past 12 Months (applied with complex sample weighting).**

Supplemental Table 1a: Odds of ED Visit for Substance Use in the Past 12 Months (applied with complex sample weighting

|  | All people (n=88,860) | | |
| --- | --- | --- | --- |
|  | Odds ratio | *p*-value | 95% C.I. |
|  |  |  |  |
| **Main Predictor** |  |  |  |
| Lifetime CLI (Yes) | **1.82** | **<0.001** | **[1.36, 2.43]** |
|  |  |  |  |
| **Demographic Covariates** |  |  |  |
| Age |  |  |  |
| 18 to 29 years old (reference) |  |  |  |
| 30 to 49 years old | **1.58** | **0.002** | **[1.20, 2.09]** |
| 50 or 50+ years old | 1.29 | 0.229 | [0.85, 1.96] |
| Male (yes) | 1.12 | 0.470 | [0.82, 1.53] |
| Race and Ethnicity |  |  |  |
| Non-Hispanic White (reference) |  |  |  |
| Non-Hispanic Black | 0.88 | 0.553 | [0.56, 1.37] |
| Non-Hispanic Others | 0.99 | 0.969 | [0.69, 1.43] |
| Hispanic | 1.17 | 0.542 | [0.70, 1.97] |
| Have one or more health insurance (Yes) | 1.18 | 0.508 | [0.71, 1.97] |
| Income |  |  |  |
| Less than $20,000 (reference) |  |  |  |
| $20,000 - $49,999 | **0.58** | **0.003** | **[0.41, 0.82]** |
| $50,000 - $74,999 | **0.35** | **<0.001** | **[0.21, 0.57]** |
| $75,000 or More | **0.39** | **<0.001** | **[0.26, 0.59]** |
| College or above (Yes) | **0.58** | **0.006** | **[0.40, 0.85]** |
| Live in metropolitan area (Yes) | 0.95 | 0.809 | [0.62, 1.45] |
| Major depressive episodes (Yes) | **1.50** | **0.034** | **[1.59, 3.18]** |
| Serious psychological distress (Yes) | **4.72** | **<0.001** | **[1.59, 3.18]** |
| Substance use disorder (Yes) | **13.44** | **<0.001** | **[7.56, 15.40]** |
|  |  |  |  |

CLI: Criminal Legal-Involved

Supplemental Table 1b: Odds of ED Visit for Mental Health in the Past 12 Months

|  | All people (n=88,860) | | |
| --- | --- | --- | --- |
|  | Odds ratio | *p*-value | 95% C.I. |
|  |  |  |  |
| **Main Predictor** |  |  |  |
| Lifetime CLI (Yes) | **1.54** | **0.001** | **[1.19, 1.99]** |
|  |  |  |  |
| **Demographic Covariates** |  |  |  |
| Age |  |  |  |
| 18 to 29 years old (reference) |  |  |  |
| 30 to 49 years old | 0.88 | 0.286 | [0.69, 1.12] |
| 50 or 50+ years old | 0.89 | 0.470 | [0.64, 1.23] |
| Male (yes) | 0.87 | 0.234 | [0.70, 1.09] |
| Race and Ethnicity |  |  |  |
| Non-Hispanic White (reference) |  |  |  |
| Non-Hispanic Black | 1.12 | 0.524 | [0.79, 1.57] |
| Non-Hispanic Others | **0.64** | **0.025** | **[0.44, 0.94]** |
| Hispanic | 1.06 | 0.719 | [0.75, 1.51] |
| Have one or more health insurance (Yes) | 1.47 | 0.060 | [0.98, 2.19] |
| Income |  |  |  |
| Less than $20,000 (reference) |  |  |  |
| $20,000 - $49,999 | **0.65** | **0.008** | **[0.48, 0.89]** |
| $50,000 - $74,999 | **0.59** | **0.025** | **[0.37, 0.93]** |
| $75,000 or More | **0.47** | **<0.001** | **[0.33, 0.66]** |
| College or above (Yes) | **0.65** | **0.008** | **[0.48, 0.89]** |
| Live in metropolitan area (Yes) | 1.03 | 0.827 | [0.77, 1.39] |
| Major depressive episodes (Yes) | **3.96** | **<0.001** | **[2.84, 5.52]** |
| Serious psychological distress (Yes) | **2.69** | **<0.001** | **[2.01, 3.58]** |
| Substance use disorder (Yes) | **2.58** | **<0.001** | **[2.07, 3.21]** |
|  |  |  |  |

Results of the logistic regression model applied with Average Treatment Effect propensity score weight (Binary outcome: treated in an emergency department for mental health)

CLI: Criminal Legal-Involved
