# Supplementary material for: Prevalence of criminal legal involvement among emergency department patients: Insights from the National Survey on Drug Use and Health 2021-2023
Source: PLoS One. 2026 Jul 8;21(7):e0351233. doi: 10.1371/journal.pone.0351233 (PMC13345442; doi:10.1371/journal.pone.0351233)
Supplement: S2 Table — Table 2a: Odds of ED Visit for Any Reason in Past 12 Months (ATT model), Table 2b: Odds of ED Visit for Substance Use in Past 12 Months (ATT model), Table 2c. Odds of ED Visit for Mental Health in Past 12 Months (ATT model). (DOCX) [file pone.0351233.s002.docx]

**S2 Table. Odds of ED Visit for Any Reason, Substance Use and Mental Health in Past 12 Months (ATT Model).**

Supplemental Table 2a: Odds of ED Visit for Any Reason in Past 12 Months (ATT model)

|  | All people (n=45,133) | | |
| --- | --- | --- | --- |
|  | Odds ratio | *p*-value | 95% C.I. |
|  |  |  |  |
| **Main Predictor** |  |  |  |
| Lifetime CLI (Yes) | **1.31** | **<0.001** | **[1.26, 1.36]** |
|  |  |  |  |
| **Demographic Covariates** |  |  |  |
| Age |  |  |  |
| 18 to 29 years old (reference) |  |  |  |
| 30 to 49 years old | 1.04 | 0.098 | [0.99, 1.10] |
| 50 or 50+ years old | **1.20** | **<0.001** | **[1.13, 1.28]** |
| Male (yes) | **0.75** | **<0.001** | **[0.72, 0.78]** |
| Race and Ethnicity |  |  |  |
| Non-Hispanic White (reference) |  |  |  |
| Non-Hispanic Black | **1.45** | **<0.001** | **[1.36, 1.54]** |
| Non-Hispanic Others | 1.02 | 0.634 | [0.94, 1.10] |
| Hispanic | 1.01 | 0.719 | [0.95, 1.08] |
| Have one or more health insurance (Yes) | **1.30** | **<0.001** | **[1.22, 1.39]** |
| Income |  |  |  |
| Less than $20,000 (reference) |  |  |  |
| $20,000 - $49,999 | **0.80** | **<0.001** | **[0.75, 0.84]** |
| $50,000 - $74,999 | **0.65** | **<0.001** | **[0.61, 0.70]** |
| $75,000 or More | **0.58** | **<0.001** | **[0.55, 0.62]** |
| College or above (Yes) | **0.57** | **<0.001** | **[0.54, 0.61]** |
| Live in metropolitan area (Yes) | **0.85** | **<0.001** | **[0.81, 0.90]** |
| Major depressive episodes (Yes) | **1.39** | **<0.001** | **[1.30, 1.59]** |
| Serious psychological distress (Yes) | **1.49** | **<0.001** | **[1.39, 1.44]** |
| Substance use disorder (Yes) | **1.38** | **<0.001** | **[1.32, 1.44]** |
|  |  |  |  |

Results of the logistic regression model applied with Average Treatment Effect of the Treated (ATT) propensity score weight (Binary outcome: treated in an emergency room for any reason)

CLI: Criminal Legal-Involved

Supplemental Table 2b: Odds of ED Visit for Substance Use in Past 12 Months (ATT model)

|  | All people (n=45,133) | | |
| --- | --- | --- | --- |
|  | Odds ratio | *p*-value | 95% C.I. |
|  |  |  |  |
| **Main Predictor** |  |  |  |
| Lifetime CLI (Yes) | **2.03** | **<0.001** | **[1.69, 2.45]** |
|  |  |  |  |
| **Demographic Covariates** |  |  |  |
| Age |  |  |  |
| 18 to 29 years old (reference) |  |  |  |
| 30 to 49 years old | **1.29** | **0.015** | **[1.05, 1.59]** |
| 50 or 50+ years old | 0.95 | 0.763 | [0.69, 1.31] |
| Male (yes) | **1.27** | **0.017** | **[1.04, 1.54]** |
| Race and Ethnicity |  |  |  |
| Non-Hispanic White (reference) |  |  |  |
| Non-Hispanic Black | 0.96 | 0.753 | [0.72, 1.27] |
| Non-Hispanic Others | 1.28 | 0.097 | [0.96, 1.70] |
| Hispanic | 1.08 | 0.573 | [0.83, 1.41] |
| Have one or more health insurance (Yes) | **1.38** | **0.027** | **[1.04, 1.84]** |
| Income |  |  |  |
| Less than $20,000 (reference) |  |  |  |
| $20,000 - $49,999 | **0.72** | **0.004** | **[0.57, 0.90]** |
| $50,000 - $74,999 | **0.44** | **<0.001** | **[0.32, 0.61]** |
| $75,000 or More | **0.50** | **<0.001** | **[0.38, 0.66]** |
| College or above (Yes) | **0.53** | **<0.001** | **[0.39, 0.73]** |
| Live in metropolitan area (Yes) | 0.97 | 0.838 | [0.76, 1.24] |
| Major depressive episodes (Yes) | **1.68** | **<0.001** | **[1.33, 2.13]** |
| Serious psychological distress (Yes) | **2.15** | **<0.001** | **[1.70, 2.72]** |
| Substance use disorder (Yes) | **7.79** | **<0.001** | **[5.93, 10.24]** |
|  |  |  |  |

Results of the logistic regression model applied with Average Treatment Effect of the Treated (ATT) propensity score weight (Binary outcome: treated in an emergency room for substance use)

CLI: Criminal Legal-Involved

Supplemental Table 2c: Odds of ED Visit for Mental Health in Past 12 Months (ATT model)

|  | All people (n=45,133) | | |
| --- | --- | --- | --- |
|  | Odds ratio | *p*-value | 95% C.I. |
|  |  |  |  |
| **Main Predictor** |  |  |  |
| Lifetime CLI (Yes) | **1.63** | **<0.001** | **[1.39, 1.91]** |
|  |  |  |  |
| **Demographic Covariates** |  |  |  |
| Age |  |  |  |
| 18 to 29 years old (reference) |  |  |  |
| 30 to 49 years old | **0.75** | **0.003** | **[0.63, 0.91]** |
| 50 or 50+ years old | **0.59** | **<0.001** | **[0.44, 0.78]** |
| Male (yes) | 0.97 | 0.761 | [0.82, 1.16] |
| Race and Ethnicity |  |  |  |
| Non-Hispanic White (reference) |  |  |  |
| Non-Hispanic Black | 1.27 | 0.060 | [0.99, 1.63] |
| Non-Hispanic Others | **1.24** | **0.114** | **[0.95, 1.61]** |
| Hispanic | 1.14 | 0.280 | [0.90, 1.44] |
| Have one or more health insurance (Yes) | **1.82** | **<0.001** | **[1.39, 2.38]** |
| Income |  |  |  |
| Less than $20,000 (reference) |  |  |  |
| $20,000 - $49,999 | **0.81** | **0.039** | **[0.66, 0.99]** |
| $50,000 - $74,999 | **0.56** | **<0.001** | **[0.42, 0.74]** |
| $75,000 or More | **0.51** | **<0.001** | **[0.39, 0.65]** |
| College or above (Yes) | **0.51** | **<0.001** | **[0.38, 0.68]** |
| Live in metropolitan area (Yes) | 0.99 | 0.917 | [0.80, 1.22] |
| Major depressive episodes (Yes) | **3.40** | **<0.001** | **[2.74, 3.08]** |
| Serious psychological distress (Yes) | **2.48** | **<0.001** | **[2.00, 3.08]** |
| Substance use disorder (Yes) | **2.14** | **<0.001** | **[1.79, 2.57]** |
|  |  |  |  |

Results of the logistic regression model applied with Average Treatment Effect of the Treated (ATT) propensity score weight (Binary outcome: treated in an emergency room for mental health)

CLI: Criminal Legal-Involved
