# Supplementary material for: Prevalence of criminal legal involvement among emergency department patients: Insights from the National Survey on Drug Use and Health 2021-2023
Source: PLoS One. 2026 Jul 8;21(7):e0351233. doi: 10.1371/journal.pone.0351233 (PMC13345442; doi:10.1371/journal.pone.0351233)
Supplement: S3 Table — Table 3a. Subgroup Analysis of Lifetime Criminal Legal-Involved Individuals on ED Visit for Substance Use in Past 12 Months. Table 3b. Subgroup Analysis of Lifetime Criminal Legal-Involved Individuals on ED Visit Mental Health in Past 12 Months. (DOCX) [file pone.0351233.s003.docx]

**S3 Table. Subgroup Analysis of Lifetime Criminal Legal-Involved Individuals on ED Visits for Substance Use and Mental Health in Past 12 Months.**

Supplemental Table 3a. Subgroup Analysis of Lifetime Criminal Legal-Involved Individuals on ED Visit for Substance Use in Past 12 Months.

| Demographic Covariates | People with arrest experience (n=133,366) | | |
| --- | --- | --- | --- |
|  | Odds ratio | *p*-value | 95% C.I. |
|  |  |  |  |
| Age |  |  |  |
| 18 to 29 years old (reference) |  |  |  |
| 30 to 49 years old | 1.71 | 0.029 | [1.06, 2.76] |
| 50 or 50+ years old | 1.02 | 0.957 | [0.49, 2.11] |
| Male (yes) | 1.21 | 0.342 | [0.81, 1.79] |
| Race and Ethnicity |  |  |  |
| Non-Hispanic White (reference) |  |  |  |
| Non-Hispanic Black | 0.68 | 0.245 | [0.36, 1.31] |
| Non-Hispanic Others | 0.86 | 0.654 | [0.44, 1.67] |
| Hispanic | 1.04 | 0.915 | [0.49, 2.24] |
| Have one or more health insurance (Yes) | **2.42** | **0.004** | **[1.35, 4.34]** |
| Income |  |  |  |
| Less than $20,000 (reference) |  |  |  |
| $20,000 - $49,999 | **0.55** | **0.014** | **[0.35, 0.88]** |
| $50,000 - $74,999 | **0.38** | **0.018** | **[0.17, 0.84]** |
| $75,000 or More | **0.35** | **0.002** | **[0.19, 0.67]** |
| College or above (Yes) | 0.72 | 0.285 | [0.39, 1.33] |
| Live in metropolitan area (Yes) | 0.86 | 0.641 | [0.46, 1.63] |
| Major depressive episodes (Yes) | **1.84** | **0.038** | **[1.04, 3.28]** |
| Serious psychological distress (Yes) | **1.72** | **0.036** | **[1.04, 2.84]** |
| Substance use disorder (Yes) | **11.06** | **<0.001** | **[6.68, 18.31]** |
|  |  |  |  |

ED: Emergency Department

Supplemental Table 3b. Subgroup Analysis of Lifetime Criminal Legal-Involved Individuals on ED Visit Mental Health in Past 12 Months.

| Demographic Covariates | People with arrest experience (n=133,366) | | |
| --- | --- | --- | --- |
|  | Odds ratio | *p*-value | 95% C.I. |
|  |  |  |  |
| Age |  |  |  |
| 18 to 29 years old (reference) |  |  |  |
| 30 to 49 years old | 0.87 | 0.488 | [0.57, 1.31] |
| 50 or 50+ years old | 1.31 | 0.378 | [0.71, 2.43] |
| Male (yes) | 1.10 | 0.701 | [0.68, 1.78] |
| Race and Ethnicity |  |  |  |
| Non-Hispanic White (reference) |  |  |  |
| Non-Hispanic Black | 0.81 | 0.488 | [0.44, 1.49] |
| Non-Hispanic Others | 0.68 | 0.327 | [0.31, 1.48] |
| Hispanic | 1.20 | 0.621 | [0.58, 2.49] |
| Have one or more health insurance (Yes) | **2.85** | **0.013** | **[1.25, 2.49]** |
| Income |  |  |  |
| Less than $20,000 (reference) |  |  |  |
| $20,000 - $49,999 | **0.57** | **0.045** | **[0.33, 0.99]** |
| $50,000 - $74,999 | 0.61 | 0.223 | [0.27, 1.37] |
| $75,000 or More | **0.32** | **0.005** | **[0.15, 0.70]** |
| College or above (Yes) | 0.42 | 0.075 | [0.16, 1.10] |
| Live in metropolitan area (Yes) | 1.26 | 0.440 | [0.69, 2.29] |
| Major depressive episodes (Yes) | **3.79** | **<0.001** | **[2.10, 6.85]** |
| Serious psychological distress (Yes) | **2.78** | **0.002** | **[1.46, 5.30]** |
| Substance use disorder (Yes) | **2.96** | **<0.001** | **[1.67, 5.22]** |
|  |  |  |  |

ED: Emergency Department
