# Supplementary material for: Prevalence of criminal legal involvement among emergency department patients: Insights from the National Survey on Drug Use and Health 2021-2023
Source: PLoS One. 2026 Jul 8;21(7):e0351233. doi: 10.1371/journal.pone.0351233 (PMC13345442; doi:10.1371/journal.pone.0351233)
Supplement: Attachment — Submitted filename: NSDUH ED Arrest PLOSOne Response to Reviewers.docx [file pone.0351233.s005.docx]

March 16, 2026

PLOS One

Ref: PONE-D-25-43064

Title: Prevalence of Criminal Legal Involvement Among Emergency Department Patients: Insights from the National Survey on Drug Use and Health 2021-2023

Authors: Vidya Eswaran, MD, Jun-Hong Chen PhD, Utsha G. Khatri, MD, Phillip L Marotta, PhD, Michael G Vaughn, PhD

Andrea K. Knittel, MD, PhD
Academic Editor, *PLOS One* Editorial Office

Dear Dr. Knittel,

We appreciate the insightful comments and suggestions provided by you and the review team for our manuscript, “Prevalence of Criminal Legal-Involvement Among Emergency Department Patients: Insights from the 2023 National Survey on Drug Use and Health”. Our team has carefully reviewed these insights and has revised our work accordingly. We also have highlighted relevant changes in the manuscript.

**Reviewer #1**: This paper presents an analysis of the relationship between emergency department utilization and recent or lifetime criminal legal involvement using data from the 2023 national survey on drug use and health. The paper presents an interesting analysis that certainly has interest and utility in our current social and policy environment. However, the analysis lacks a certain degree of clarity in its methodology—specifically related to matters of sampling and measurement—and its significance or impact for practitioners and policymakers in general.

- Response: Thank you for this comment and for allowing us the opportunity to make edits to address concerns related to clarity in methodology and significance to practitioners and policy makers.

**Comment 1**: To begin with the significance or impact of this analysis, the utility of the author's findings to policy or practice is not sufficiently articulated in the manuscript. Throughout the abstract as well as the manuscript itself (including on page 4 in the introduction, page 5 in the introduction, and page 15 and 16 in the discussion), the authors repeatedly mention the need for emergency medicine providers to better understand and address or to better identify and treat the specific needs of people presenting to an emergency department with a history of criminal legal involvement. However, what those specific patient needs or provider actions that might be is never clearly articulated in the draft, and I am struggling to imagine what particular, individualized intervention any healthcare practitioner could take that would target an underlying structural driver of the surface level biological or psychological concern they are there to treat. In other words, how should a healthcare provider treat a person presenting to the emergency department with a substance use related issue any differently based on their history (or lack thereof) of criminal legal involvement?

I don't mean to throw the baby out with the bathwater and say that healthcare providers have nothing to offer in terms of structural supports to the people that they serve. Nevertheless, the authors failed to clarify how the needs of patient presenting to an emergency department with a mental illness or substance use related concern would be fundamentally different according to that individual's current or prior criminal legal involvement. The practical details of this distinction is of the utmost importance to the argument that the authors appear to be trying to make. Yet it is glaringly absent from the paper. Only in the discussion section do the authors begin to theorize how criminal legal involvement of any kind might have a direct impact on the likelihood of someone's presentation to the emergency department, identifying lasting trauma from criminal legal involvement as a driver of behavioral health concerns or lack of access to basic life needs and financial resources as a driver of emergency department utilization. (Importantly, the authors don't explicitly identify these as plausible mechanisms explaining their findings, but they absolutely should.) Yet those plausible mechanisms don't to address at all how people with past criminal legal involvement might somehow present differently to an emergency department or how the needs they present with might be fundamentally different or how they should be addressed in some specific, targeted way. In other words, it's not at all clear how healthcare providers are supposed to treat people with criminal legal involvement differently or understand their problems through some different framework.

On page 16 the authors do make a couple of suggestions about what that could look like in a practical sense, but those suggestions are limited to a brief mention of connecting patients with reentry focused resources, which—I feel quite strongly should be noted is not a healthcare provider's job; That is the job of jails and prisons and existing state-run reentry services—and building collaborations and supports across hospital departments, which would be a benefit to all patients presenting with behavioral health issues not just those with criminal legal involvement. So, in the end, it's not at all clear what practitioners or policymakers are supposed to do with the information provided in this manuscript especially insofar as the authors are focusing on individual level health care practices more so than structural changes or population level policy changes that might begin to address the problematic relationships their analysis identifies.

**Response**: Thank you for this insightful comment. We agree with the reviewer that further development of next steps and the clinical impact of these findings is needed. At the individual-level, we believe that many ED clinicians are likely unaware that CLSI impacts such many ED patients. CLSI is a known risk factor for short and long term health outcomes and is associated with premature mortality. It is important that clinicians are educated on these risks and to consider them when developing treatment plans and referral prioritization. Further, while in recent years focused training on the provision of trauma-informed care has become more common, we believe it vitally important for clinicians to be aware of the harms caused by incarceration and how it may impact their interaction with patients. We have included more specific examples of ED based interventions and included ED social workers, case managers and hospital and health care leaders as important stakeholders when planning interventions to address the needs of patients with CLSI. Selected text has been included below highlighting these changes.

- Page 7 Line 111: *Understanding which patients are more likely to rely on ED care, and why, may help inform more tailored and equitable ED-based interventions.*

*Although patients with CLI experience higher risks for adverse health outcomes, it remains unclear whether and how knowledge of CLI adds meaningful information beyond the identification of SUD, psychiatric disease, or other social needs commonly encountered among ED patients. While social determinant screening is becoming more common in health care settings ^31–33^, screening for CLI is rare, and its potential utility in ED care has not been well defined. The existing literature on ED utilization among people with CLI focuses on cohorts with known and recent incarceration, often identified through administrative data linkages. ^18,27,34^ Less is known about the prevalence of lifetime CLI among the broader ED population, or whether individuals with a history of CLI, regardless of how recent, display different patterns of ED utilization. Without such data, designing targeted interventions and understanding the role ED settings can play in addressing substance use and mental health needs in this population remains challenging*

- Page 19 Line 349: *There is growing understanding of the importance of competency in the provision of trauma informed care among ED clinicians.^55^ Given the significant trauma experienced by many while incarcerated,^56^ that mistrust of health care systems and providers persist after release from carceral facilities, and that many people who were formally incarcerated report bias from healthcare providers,^57,58^ knowing that 20% of ED patients have experienced incarceration can be helpful in motivating clinicians to be prepared to deliver trauma-informed care. Practically, this could include lectures to medical students and trainees about mass incarceration and the short- and long-term health impacts, including personal or familial incarceration in lectures related to social determinants of health, inviting individuals with lived experience of incarceration to share their experience in lectures or small groups, and frequent training and skills building in best practices related to the provision of trauma informed care.*

*Incarceration has been associated with increased risk of adverse medical and behavioral health outcomes and premature mortality.^59–62^ It is important that ED clinicians are aware of these risks and consider them when developing treatment plans and prioritizing patient referrals for outpatient care. Beyond, individual level care, awareness of high prevalence of CLI among ED patients is important for health systems when considering interventions and resource development. EDs have long been centers of implementation for public health screening and intervention.^63–65^ Multiple ED-based programs exist to address basic needs such as food and housing insecurity, for example.^66–68^ Universal screening of ED patients for HIV have improved rates of diagnoses and linkage to care.^69^ Given proposed and recent changes to Medicaid policy, more individuals may lose access to Medicaid and thus rely on EDs for care beyond their acute, emergent needs.^70^ Awareness of CLI among a large portion of ED patients should inspire careful consideration by ED and hospital leadership to consider how ED staff and health care systems can best support this population. ED-based social workers and case managers may be able to connect patients with re-entry focused resources within their community to provide continued support beyond the hospital walls. Medical-Legal partnerships^71,72^ could be developed to bring comprehensive social services to patients being seen in the ED. Collaboration between addiction medicine, mental health clinics and EDs could include warm handoffs between clinical sites, and assistance with implementation of protocols to facilitate the provision of medications for substance use disorders in the ED to patients who may otherwise have limited access to outpatient healthcare, especially given known disparities in access to standard of care treatments among those with CLI. The ED could be a meaningful point of intervention and partner to prevent adverse health outcomes, and potentially recidivism and re-incarceration as well.*

**Comment 2**: With regard to measurement and method, I understand that the authors are limited to the indicators that were already included in the national survey on drug use and health. However, it seems to me that the problems with using a measurement like arrest and booking as an indicator for criminal legal involvement should be obvious and openly discussed both in the plan for analysis and in the limitations section of the paper. What arrest and booking is able to measure is, in a way, NEW episodes of criminal legal involvement. Or perhaps even ongoing episodes of criminal legal involvement. I'm thinking here of someone who has been on community supervision for 18 months after release from a period of incarceration has not been arrested or booked during that time period. That person absolutely has been subject to criminal legal involvement during the past 12 months but that would not be identified by the measures used in the study. Thus one major problem with this measurement is the fact that people who are under community supervision or some other degree of criminal legal involvement that has not resulted in an arrest on a booking in the past 12 months cannot be identified and assigned to the treatment group as appropriate.

**Response:** Thank you for this comment. We agree that the use of arrest or booking as a marker for past-year CLSI is limited for the reasons you have mentioned. We include lifetime CLSI as a key exposure in our study partly for this reason. We have added language to the text to further highlight this limitation.

- Page 10 Line 164: *Past year CLI was defined as an answer of greater than 0 to the question “Not counting minor traffic violations, how many times during the past 12 months have you been arrested and booked for breaking a law?” and serves as a marker of new involvement with the criminal legal system.*
- Page 23 Line 406: *Fourth, the use of ‘arrest and booking’ to define CLI, especially in the past-year, limits CLI to new episodes of criminal legal involvement and fails to capture those under community supervision who, while still ‘involved’ with the system, may not have been arrested or booked during the prior twelve months. The use of lifetime CLI is intended to more broadly include this population.*

**Comment 3**: Further, the sampling limitations that are inherent in the national survey on drug use and health methodology need to be articulated in the methods section, not just the limitations section and the impact of those sampling challenges on the results that the authors have produced and need to be discussed much more thoroughly and clearly in the paper. Lots of other scholars have addressed the deeply ironic sampling challenges with this survey, so the authors are well within their rights to cite out to other works that have already produced this kind of analysis of the surveys sampling structure and the problems it injects into its data. But it has to be brought up explicitly ahead of the analysis in paper.

**Response**: Thank you for this comment. We have included the following text in the Methods section of the manuscript:

- Page 9 Line 148: *Given the sampling strategy of NSDUH it is important to contextualize the study understanding that housing insecure and currently incarcerated or otherwise institutionalized individuals are not included, especially given that both those these social risks are associated both with increased risk of incarceration and ED use.^27,36,37^*

**Comment 4:** In the opening lines of the paper, the authors make reference to “Western countries.” This term can be a bit problematic and it's usually best to avoid using it whenever possible. If the authors simply mean that the United States has incarcerated more people than other countries in Europe and North America, say that. If the author is mean that the United States has the highest incarceration rate of countries with GDP above a certain amount, say that. It's always preferable to just say what you mean instead of glossing it with squishy and problematic terms like “western.”

**Response:** Thank you for this comment. We have amended this statement as noted below.

- Page 4 Line 55: *The United States (US) incarcerates more of its citizens than any other high-income country, with incarceration rates exceeding those of peer nations*

**Comment 5**: There seems to be a citation missing at the end of the second sentence, ending in the phrase “drug related crime.”

**Response**: Thank you for this comment. References have been added.

**Comment** **6**: The authors use the term prisoners at the end of the first paragraph. This term is not preferred.

**Response**: Thank you for this comment. We have changed the text as noted below:

- Page 5 Line 79: *It is estimated that 30-45% of those incarcerated in prison have at least one chronic medical condition or disease, ^5,6^ and many report current or prior infectious disease history, such as HIV or Hepatitis C.^5^*

**Comment 7**: On page 4 the authors refer to other sources of care that are decreasing in the United States. It would be helpful to state explicitly what those are.

**Response**: Thank you for this comment. We have amended the text as noted below:

- Page 6 Line 105: *As EDs increasingly become an important access point to identify and treat substance use disorders ^26^, and in an era within the US where access to other sources of care, such as inpatient psychiatric facilities and safety-net clinics, may be decreasing,^27,28^ their role in addressing health disparities related to CLI is gaining attention. ^29,30^*

**Comment 8:** The authors do a good job in the measures section of explaining what the term booked means, but a brief explanation of how that connects to other forms of criminal legal involvement (as in being booked is the gateway to other forms of criminal legal involvement like prosecution, drug court, community service, probation, etc.) would be helpful.

**Response**: Thank you for this comment. We have amended the text as noted below:

- Page 10 Line 162: *Those who are booked may subsequently be prosecuted and incarcerated, referred to drug courts or community service, or referred to probation and other community supervision.*

**Comment 9:** On page 14 the authors state that their findings suggest associations between criminal legal involvement and emergency department use persist over time. In this context, the term persists suggests a temporal model of causality between criminal legal involvement and emergency department utilization that is not supported by this study and seems plausible but unlikely given the other bodies of evidence that exist, many of which the authors are already referring to. Put another way, many socioeconomic factors themselves are predictive of both criminal legal involvement and emergency department utilization, so if the authors want to suggest that an effect of criminal legal involvement persists over time they need to establish that something is an effect of criminal legal involvement. Whether through their own data or through reference to other literature, they have not done so here.

**Response**: Thank you for this comment. We have amended the text as noted below:

- Page 17 Line 315: *our findings suggest that the association between CLI and ED exists beyond this known high-risk period*

**Comment 10:** The study mentioned in the discussion that shows very low levels of criminal legal involvement among emergency department patients via an analysis of electronic health records seems important context for the introduction.

**Response**: Thank you for this comment. We have included the following in the introduction:

- Page 8 Line 126: *Efforts to characterize CLI among ED populations using natural language model analysis of electronic medical record notes describe low prevalence of CLI but have not been robustly validated.*

**Reviewer #2:** This is an interesting manuscript on an important topic which is the impact of criminal justice involvement in use of emergency departments.

**Comment 1:** There are a few areas where the grammar needs to be reviewed and some spelling errors (see line 135 "non-Hispanic Back should be "Black").

**Response**: Thank you for this comment. We have proofread and corrected errors including the error noted in the comment.

**Comment 2**: The authors effectively highlight the intersection of incarceration, substance use, mental health, and healthcare access, particularly in ED settings. The justification for focusing on lifetime CLI rather than only recent involvement is persuasive and adds depth to the study's relevance.

**Response**: Thank you for this comment.

**Comment 3**: The dataset is nationally representative but has its drawbacks which are discussed in the limitations section.

**Response**: Thank you for this comment.

**Comment 4**: The methodology is robust, employing descriptive statistics, logistic regression, and propensity score weighting to account for confounding variables. The paper would benefit from a little more discussion of the use of both logistic regression and propensity score analysis, and how they complement each other and why both are needed, especially with respect to Table 4.

**Response**: Thank you for this comment. We have amended our methods to account for reviewers suggestions and have removed propensity score weighting for Table 4, and instead ensured the use of complex sample weighting and have limited propensity score analysis to the main analyses in Supplemental Table 2a-c.

Our main purpose of applying complex sample weighting is to show nationally representative results. We chose to also perform propensity score weight (PSW) to minimize the demographic feature difference between the control population and the treatment population (e.g., people with CLI). We reduce sample selection bias by minimizing the demographic feature difference between the control and the treatment population and are thus able to enhance causal inference (though understanding that this does not mean fully causal inference as there are always unobservable differences). There are two forms of PSW when considering binary status treatment: Average Treatment Effect (ATE) and Average Treatment Effect on the Treated (ATT). We chose to report ATT in supplemental tables to focus on the population with CLI.

Comment 5: While it is not a novel finding that formerly incarcerated individuals access the ED for healthcare, and that these are important social determinants of health, the manuscript adds incrementally to our knowledge of the impact of incarceration on healthcare-seeking behavior.

**Response**: Thank you for this comment.

**Comment 6**: The authors advocate for screening and tailored interventions in EDs, which is a logical extension of their findings. However, as the authors note, that could be a heavy lift as most EDs do not collect that information and the authors themselves state that it is not clear or established how data on previous incarceration would be collected in that setting.

**Response**: Thank you for this comment. We have expanded our discussion on screening as noted below.

- Page 21 Line 382: *EDs routinely screen patients for a variety of conditions including domestic violence, suicidality, substance use disorders, travel history and risk of falls.78 Before wholescale screening for CLI is implemented in EDs, however, it will be important to understand the acceptability of ED-based screening among CLI populations and to thoughtfully consider how and when such screening would take place to minimize undue burden on ED staff. Further, patients with a history of CLI have reported experiencing biased care from health care providers 62,79. Data from ED nurses, advanced practice providers and physicians show the belief that incarcerated patients receive differential care 80,81. Data from Internal Medicine healthcare providers demonstrate that clinicians are unsure on how to ask about patients’ CLI 82. We encourage involvement of individuals with CLI history and community organizations whose work center these populations when considering how to screen patients and how to act on positive screens to ensure screening does not increase actual or perceived discrimination by health care providers. Partnering with local correctional health authorities to facilitate data exchange could also help clinicians in and out of carceral settings understand the health needs of the patients they share.*

**Comment 7**: Finally, the paper could use additional discussion on whether findings from these data are truly generalizable.

**Response:** Thank you for this comment. We have included more information about generalizability to the discussion as noted below.

- Page 23 Line 414 *Given this is a nationwide sample, regional differences in prevalence likely exist between urban, suburban and rural settings and by local/state jurisdiction. Further work is needed to examine differences between settings such that health care organizations, carceral facilities and policymakers can identify where best to target resources.*

**Reviewer #3**: Authors: This study uses a retrospective cohort design from publicly available nationally representative sample to look at criminal legal system involvement among those with a past year emergency department visit. This is a novel question that has not been previously undertaken to my knowledge. It can be an important question, given that health disparities are well described amongst people with justice involvement, compared with the general population, and identifying these individuals may provide an opportunity for targeted health system interventions such as linkage to peers, medical legal partnerships, rapid housing and targeted primary care programs.

**Comment 1**: This manuscript will benefit from more clearly laying out the current state of knowledge on this topic and what specifically this study adds, as well as the specific research question. I do believe that with moderate edits, this manuscript can contribute meaningfully to the literature on healthcare delivery and quality of community care delivery for previously incarcerated people, especially where they most often access care- in the emergency department.

**Response:** Thank you for this comment. We have worked to more clearly outline the current state of knowledge and gaps in the literature as you have recommended as noted below (see response to comments 4 and 5).

**Comment 2:** Title: Suggest remove hyphen from title.

**Response:** Thank you for this comment. We have amended the title as suggested.

**Comment 3:** Abstract: Not clear to the average reader why this is a problem. No mention of health risks, so why should EDs care if there is past CLI? Opportunity to more clearly state the problem in the intro. Advise reorder the results for ED visits among CLI line 45-46 in abstract so that consistent with reporting order in the sentences prior. It is otherwise more confusing for the reader.

**Response:** Thank you for this comment. We have amended the abstract to include more justification in the introduction and re-ordered the results as noted below.

- Page 2 Line 34: *Criminal legal involvement (CLI) is a known predictor of short- and long-term adverse health outcomes, and those with CLI often use emergency departments (EDs) to access health care. However, the prevalence of CLI among ED patients is unknown. We sought to assess lifetime and past year CLI among those reporting an ED visit for any reason, for substance use and for mental health, and predictors of ED use among those with CLI to inform efforts to establish ED-based screening and interventions.*
- Page 2 Line 45: *These numbers rose to 44% (lifetime CLI) and 15% (past year CLI) for substance use related ED visits and 29% (lifetime CLI) and 8% (past year CLI) for mental health related ED visits.*

Introduction:
**Comment 4**: As currently written it is challenging to follow and could be tightened. The authors present a broad overall epidemiology of incarceration in the US, of substance use disorder treatment for those with CLI, then social determinants, and then to the role of the ED in the life of people with CLI. I suggest really tightening and getting to the health risks right away and then the way this population uses the ED and why identifying CLI might be helpful in the ED. Why is it any more helpful that just identifying the SUD or the mental health condition? These patients are also often unstably housed and with many social determinant needs. Again, why is this helpful? What might the ED do different for this population than any other population with SUD or mental health conditions? Line 78-79 mentions this vaguely, but being more specific would be helpful in setting up the value of this line of inquiry.

**Response**: Thank you for this detailed and insightful comment. We appreciate the recommendations and have amended the introduction as noted below.

- Page 4 Line 55: *The United States (US) incarcerates more of its citizens than any other high-income country, with incarceration rates exceeding those of peer nations.^1^ In 2022 1.8 million people were incarcerated in US prisons or jails. ^2^ A history of incarceration has been associated with increased individual risk of chronic physical and mental health conditions even after release, including cardiovascular disease, chronic lung disease, mental health conditions, substance use disorders, and hearing impairment.^3–6^ Communities in which incarceration is more common are more likely to see the spread of communicable disease such as sexually transmitted infections.^7–9^ Parental incarceration can also have long-term impact on the physical and mental health of their children.^10,11^ Post-incarceration, individuals are more likely to use acute settings such as Emergency Departments (EDs) than primary care settings not only in the immediate period following release, but long-term as well.^12–15^*
- Page 6 Line 105: *As EDs increasingly become an important access point to identify and treat substance use disorders, ^37^ and in an era within the US where access to other sources of care, such as inpatient psychiatric facilities and safety-net clinics, may be decreasing,^38,39^ their role in addressing health disparities related to CLI is gaining attention. ^40,41^ Between 2018-2019 and 2020-2021, the rate of ED visits for substance use disorder increased from 74.4 to 103.8 per 10,000 population. Even so, inequities in provision of MOUD from EDs have been described, ^42^ and opportunities to identify and treat more patients remain. ^43^ Understanding which patients are more likely to rely on ED care, and why, may help inform more tailored and equitable ED-based interventions.*

*Although patients with CLI experience higher risks for adverse health outcomes, it remains unclear whether and how knowledge of CLI adds meaningful information beyond the identification of SUD, psychiatric disease, or other social needs commonly encountered among ED patients. It is known, however that history of CLI can limit opportunities to access housing and food benefits,^44,45^ and it is likely that those with a history of CLI could benefit from tailored interventions which are trauma-informed and accessible based on their history. While social determinant screening is becoming more common in health care settings, ^44–46^ screening for CLI is rare, and its potential utility in ED care has not been well defined. The existing literature on ED utilization among people with CLI focuses on cohorts with known and recent incarceration, often identified through administrative data linkages. ^12,40,47^ Less is known about the prevalence of lifetime CLI among the broader ED population, or whether individuals with a history of CLI, regardless of how recent, display different patterns of ED utilization. Efforts to characterize CLI among ED populations using natural language model analysis of electronic medical record notes describe low prevalence of CLI but have not been robustly validated.^48^ Without such data, designing targeted interventions and understanding the role ED settings can play in addressing substance use and mental health needs in this population remains challenging.*

**Comment 5**: Additionally would be most helpful to summarize the literature on ED use for this population and what the gaps really are. As currently written this introduction does not present in detail what are the past findings on ED use for this populations and what are the limitations of the current body of research. This would set up for a stronger final paragraph where the authors can more clearly and specifically define the research question. For example, the first author has previously published work that established that frequent ED visits are associated with subsequent jail incarceration and that the ED is a place for intervention to prevent incarceration- how does this study build off of that work? Is the question what is spelled out in second sentence of the abstract? Is this really what you sought to assess?: “We sought to assess lifetime and past year CLI among those reporting an ED visit for any reason, for substance use and for mental health.” Having read through the manuscript this does not appear to be the primary question, your methods and results sections focus much more on differential use of the ED for people with and without CLI, so is the question how people with CLI use the ED or is the question what percent of people going to an ED have CLI? If your question is as stated, then Table 1 makes sense, but then why Table 2 and related analysis? What does this study add that data linkage studies that have linked jail and ED records have not addressed (see Easter et al, Journal of Correctional Healthcare, 2023; Humphreys, JUH, 2017) and what does this add beyond the work of Joseph Frank et al in JGIM that you cited but really didn’t expand upon- they did a similar analysis, why is this study needed and different?

**Response**: Thank you for this comment. We have updated our introduction paragraph to more completely tie in prior research and to show why our study is novel and necessary to move the needle forward in the development and implementation of ED-based screening and interventions for those with CLI history. Notably, the Frank study mentioned by the reviewer uses survey data from 2008-2011, we include more recent data given the significant changes in the health care landscape since that time. Other data start with a cohort of individuals with known CLI and assess ED utilization. This does not address the prevalence of CLI among the ED population as a whole, which is important for ED leadership to understand in order to prioritize the implementation of interventions for this population compared to others seeking ED care.

- Page 4 Line 64: *Prior research has shown that after release from carceral facilities, those who have been incarcerated preferentially access health care in EDs. Data from 2008-2011 regrading self-reported past-year incarceration, parole or probation is associated with increased odds of hospitalization and ED utilization and disproportionate hospital expenditures.^12^ In linked data analysis, half of all individuals living with HIV who were released from jail in one county had an ED visit within the first year and had 5x higher proportion of ED visits for substance use or mental health compared to the general population.^13^Another single-jurisdiction study of older adults released from jail showed similarly high rates of ED use among older adults in the 6 months post-release.^14^ Individuals released from jail are also more likely to visit EDs frequently, and frequent ED use was also associated with frequent jail bookings, as shown in two single-county analyses.^15,16^ Long-term, individuals who have experienced incarceration are also less likely to have a usual source of care or receive preventive services compared to those who have not been incarcerated.^17^*
- Page 7 Line 120: *The existing literature on ED utilization among people with CLI focuses on cohorts with known and recent incarceration, often identified through recent administrative data linkages or uses survey data from pre-Affordable Care Act implementation, which has significantly changed rates of health care utilization. ^12,14–16^ Less is known about the prevalence of lifetime CLI among the broader ED population, or whether individuals with a history of CLI, regardless of how recent, display different patterns of ED utilization. Efforts to characterize CLI among ED populations using natural language model analysis of electronic medical record notes describe low prevalence of CLI but have not been robustly validated.^51^ Without such data, designing targeted interventions and understanding the role ED settings can play in addressing substance use and mental health needs in this population remains challenging.*
- Page 2 Line 36: *We sought to assess lifetime and past year CLI among those reporting an ED visit for any reason, for substance use and for mental health, and predictors of ED use among those with CLI to inform efforts to establish ED-based screening and interventions.*
- Page 8 Line 131: *To address this gap in the literature, our study aimed to characterize the prevalence of lifetime CLI among individuals reporting emergency department utilization in the prior year and by examining whether lifetime CLI is associated with differential patterns of ED utilization. Using nationally representative data, we assessed the prevalence of lifetime CLI among individuals reporting ED visits for any reason, as well as ED visits related to substance use and mental health. Furthermore, we examined whether lifetime CLI was associated with an increased odds of ED use after accounting for sociodemographic and health-related factors. While our primary focus was on lifetime CLI, we also evaluated associations between past-year CLI and ED use to provide context regarding the temporal relationship of incarceration and health care use when considering policy solutions.*

Methods:

Comment 6: Why did the authors not include the question about probation or parole in defining CLI? Past studies have. If there was a strong rationale, would explicate here. If not including this, why not call this variable what it is, which is prior incarceration, not the broader conceptualization of criminal legal system involvement which typically includes incarceration and community supervision through probation and parole.

**Response:** Thank you for this comment. We chose to use ‘arrested or booked’ as the exposure of interest to specifically assess the interconnection between arrest and ED use, understanding that what can happen to an individual post arrest or booking is quite variable. Given that most individuals in community supervision would have been arrested or booked at some point in the lifetime, we chose our primary exposure to mark the entry-point into the criminal legal system. We have amended our language in the manuscript to more clearly highlight this as recommended by a previous reviewer and as noted below.

- Page 10 Line 164: *Past year CLI was defined as an answer of greater than 0 to the question “Not counting minor traffic violations, how many times during the past 12 months have you been arrested and booked for breaking a law?” and serves as a marker of new involvement with the criminal legal system.*
- Page 23 Line 406: F*ourth, the use of ‘arrest and booking’ to define CLI, especially in the past-year, limits CLI to new episodes of criminal legal involvement and fails to capture those under community supervision who, while still ‘involved’ with the system, may not have been arrested or booked during the prior twelve months, suggesting prevalence of past year CLI may in truth be higher than our results suggest. The use of lifetime CLI is intended to more broadly include this population*

**Comment 7**: Also, why the decision to use one year of NSDUH data instead of combining multiple years to increase sample of people with recent CLI and different ED use?

**Response:** Thank you for this comment. We chose to include data from 2023 such that the most recent year of NSDUH data available at the time of analysis could be reviewed. We have since expanded our analysis to include years 2021-2023 to increase our sample size.

**Comment 8**: Why did the authors feel that propensity score matching was necessary when past studies using NSDUH with this population have not employed this approach, especially given that there are not that many other variables to control for between CLI and non-CLI? It would be add to your methods if you describe the need for this methodology more and what deficits it responds to in prior studies using NSDUH.

Thank you for this comment. We have amended our methods to focus primarily on complex sample weighting however include propensity score weights in our supplemental table for the main analyses. We chose to also perform propensity score weight (PSW) to minimize the demographic feature difference between the control population and the treatment population (e.g., people with CLI). We reduce sample selection bias by minimizing the demographic feature difference between the control and the treatment population and are thus able to enhance causal inference (though understanding that this does not mean fully causal inference as there are always unobservable differences). There are two forms of PSW when considering binary status treatment: Average Treatment Effect (ATE) and Average Treatment Effect on the Treated (ATT). We chose to report ATT in supplemental tables to focus on the population with CLI.

Page 13 Line 218: *For causal inference that accounts for potential selection bias in CLI, we applied propensity score weighting ^62,63^ to ensure the treatment group (i.e., people with CLI) and the control group (i.e., people without CLI) had similar demographic characteristics and similar baseline psychological distress (i.e., no significant difference between the control and exposed groups).*

*To assess primary and secondary outcomes by CLI, we employed average treatment effects using propensity score weight. To further reduce estimation bias, we isolated the effects of CLI from demographic features using doubly robust estimation approach ^64,65^. Doubly robust estimation is a statistical method used in causal inference to estimate the effect of an intervention or treatment on an outcome variable. While propensity score weight aims to balance the covariates between different subgroups, the robust estimation approach helps differentiate out the covariate effects ^66^.*

**Comment 9**: Discussion: Overall the discussion feels more compelling than the introduction and gets the reader to make sense of the results. I think one thing that could be teased out more is what health systems would even do about any prior lifetime CLI exposure? The argument to identify recent release or recent CLI is certainly more supported by the literature and feels more actionable.

**Response**: Thank you for this comment. We have included information in the discussion regarding the need for training in trauma-informed care and the long term health impacts of incarceration among ED clinicians and how health systems and ED leadership should respond in light of our findings. The updated text is included below.

- Page 19 Line 349: *There is growing understanding of the importance of competency in the provision of trauma informed care among ED clinicians.^74^ Given the significant trauma experienced by many while incarcerated,^75^ that mistrust of health care systems and providers persist after release from carceral facilities, and that many people who were formally incarcerated report bias from healthcare providers,^76,77^ knowing that 20% of ED patients have experienced incarceration can be helpful in motivating clinicians to be prepared to deliver trauma-informed care. Practically, this could include lectures to medical students and trainees about mass incarceration and the short- and long-term health impacts, including personal or familial incarceration in lectures related to social determinants of health, inviting individuals with lived experience of incarceration to share their experience in lectures or small groups, and frequent training and skills building in best practices related to the provision of trauma informed care.*

*Incarceration has been associated with increased risk of adverse medical and behavioral health outcomes and premature mortality.^7,78–80^ It is important that ED clinicians are aware of these risks and consider them when developing treatment plans and prioritizing patient referrals for outpatient care. Beyond, individual level care, awareness of high prevalence of CLI among ED patients is important for health systems when considering interventions and resource development. EDs have long been centers of implementation for public health screening and intervention.^81–83^ Multiple ED-based programs exist to address basic needs such as food and housing insecurity, for example.^84–86^ Universal screening of ED patients for HIV have improved rates of diagnoses and linkage to care.^87^ Given proposed and recent changes to Medicaid policy, more individuals may lose access to Medicaid and thus rely on EDs for care beyond their acute, emergent needs.^88^ Awareness of CLI among a large portion of ED patients should inspire careful consideration by ED and hospital leadership to consider how ED staff and health care systems can best support this population. ED-based social workers and case managers may be able to connect patients with re-entry focused resources within their community to provide continued support beyond the hospital walls. Medical-Legal partnerships^89,90^ could be developed to bring comprehensive social services to patients being seen in the ED. Collaboration between addiction medicine, mental health clinics and EDs could include warm handoffs between clinical sites, and assistance with implementation of protocols to facilitate the provision of medications for substance use disorders in the ED to patients who may otherwise have limited access to outpatient healthcare, especially given known disparities in access to standard of care treatments among those with CLI. The ED could be a meaningful point of intervention and partner to prevent adverse health outcomes, and potentially recidivism and re-incarceration as well.*

**Comment 10:** 281-283- unclear what this sentence means

**Response:** We have removed this sentence.

**Comment 11:** 296-298- in fact, data from NSDUH suggest that the increase in Medicaid post ACA expansion was not associated with increased engagement among people with CLI. (Howell et al, Psych Services, 2023), so this would be helpful to add here and contextualize your findings based on these past findings.

**Response:** Thank you for this comment. We have included this citation to contextualize our results as noted below.

- Page 19 Line 337: *Other data have shown that while policy efforts related to Medicaid expansion lead to increased insurance coverage among those with past-year CLI, there was no change in the receipt of treatment for substance use disorders or mental health conditions.^73^ This finding warrants further exploration to understand whether health insurance coverage translates into actual health care access and use of preventive or longitudinal care, especially the care needed for managing substance use and co-occurring condition, and if not, to understand what other barriers these populations face to accessing outpatient care.*

**Comment 12**: Table 2: N should be in first row for CLI vs no CLI.

**Response:** Thank you for this comment. We have included this in our updated table.

**Reviewer #4**:

Introduction
Comment 1: - this section could focus on statements which directly support the scientific question at hand. The second and third paragraphs do not seem to fit where they have been placed.

**Response:** Thank you for this comment. We have reorganized the introduction based on reviewer comments. Specifically we have moved the cited paragraphs further down in the introduction.

**Comment 2**: - The problems created by the MIEP go much further than access to health insurance - it creates a siloed carceral health system and prevents funding for transitional services. This is a nice review of the matter, it would be worthwhile to update the sentence beginning line 71 on page 2: <https://pmc.ncbi.nlm.nih.gov/articles/PMC11281997/>

**Response:** Thank you for this comment. We have expanded the sentence as noted below.

- Page 6 Line 93: *Barriers to timely outpatient care include disruptions in health insurance due to the Medicaid Inmate Exclusion Policy which limits oversight of the medical care offered by carceral facilities and leads to the suspension or termination of Medicaid when a person is incarcerated, though the proposed Medicaid Reentry Act may alleviate this barrier in the future.^35,36^*

**Comment 3:** the first paragraph on page 5, is not very scholarly. While there are many gaps in knowledge about CLI and ED utilization, there is a not a dearth of studies on the matter either. Moreover, many of the studies that you cite use the same data source as this paper, so more clearly identifying the precise gap in knowledge would strengthen the scientific rigor of this introduction.

**Response:** Thank you for this comment, we have included more detailed information on the studies and revised the paragraph mentioned by this reviewer.

- Page 4 Line 64: *Prior research has shown that after release from carceral facilities, those who have been incarcerated preferentially access health care in EDs. Data from 2008-2011 regrading self-reported past-year incarceration, parole or probation is associated with increased odds of hospitalization and ED utilization and disproportionate hospital expenditures.^12^ In linked data analysis, half of all individuals living with HIV who were released from jail in one county had an ED visit within the first year and had 5x higher proportion of ED visits for substance use or mental health compared to the general population.^13^Another single-jurisdiction study of older adults released from jail showed similarly high rates of ED use among older adults in the 6 months post-release.^14^ Individuals released from jail are also more likely to visit EDs frequently, and frequent ED use was also associated with frequent jail bookings, as shown in two single-county analyses.^15,16^ Long-term, individuals who have experienced incarceration are also less likely to have a usual source of care or receive preventive services compared to those who have not been incarcerated.^17^*
- Page 7 Line 113: *Although patients with CLI experience higher risks for adverse health outcomes, it remains unclear whether and how knowledge of CLI adds meaningful information beyond the identification of SUD, psychiatric disease, or other social needs commonly encountered among ED patients. It is known, however that history of CLI can limit opportunities to access housing and food benefits,^47,48^ and it is likely that those with a history of CLI could benefit from tailored interventions which are trauma-informed and accessible based on their history. While social determinant screening is becoming more common in health care settings, ^49–51^ screening for CLI is rare, and its potential utility in ED care has not been well defined. The existing literature on ED utilization among people with CLI focuses on cohorts with known and recent incarceration, often identified through recent administrative data linkages or uses survey data from over 15 years ago, during which time significant changes in health care policy have been made, such as Medicaid expansion and the Affordable Care Act. ^12,14–16^ Less is known about the prevalence of lifetime CLI among the broader ED population, or whether individuals with a history of CLI, regardless of how recent, display different patterns of ED utilization. Efforts to characterize CLI among ED populations using natural language model analysis of electronic medical record notes describe low prevalence of CLI but have not been robustly validated.^52^ Without such data, designing targeted interventions and understanding the role ED settings can play in addressing substance use and mental health needs in this population remains challenging.*

*To address this gap in the literature, our study aimed to characterize the prevalence of lifetime CLI among individuals reporting emergency department utilization in the prior year and by examining whether lifetime CLI is associated with differential patterns of ED utilization. Using nationally representative data, we assessed the prevalence of lifetime CLI among individuals reporting ED visits for any reason, as well as ED visits related to substance use and mental health. Furthermore, we examined whether lifetime CLI was associated with an increased odds of ED use after accounting for sociodemographic and health-related factors. While our primary focus was on lifetime CLI, we also evaluated associations between past-year CLI and ED use to provide context regarding the temporal relationship of incarceration and health care use when considering policy solutions.*

Methods
**Comment 4:** Is there any reason that the authors did not combine this data set with 2022 or 2021? The N for several outcome groups is very small, limiting interpretation of these results. Datasets can be combined per NSDUH recommended procedures (though I believe 2020 should not be combined so you may be limited is 2021-2023.

**Response:** Thank you for this comment. We chose to include data from 2023 such that the most recent year of NSDUH data available at the time of analysis could be reviewed. We have since expanded our analysis to include data from 2021-2023 to increase our sample size.

**Comment 5:** Given the outcome is healthcare utilization, and variables on other comorbid health conditions is available in the dataset, it seems appropriate to control for these as well.

**Response**: Thank you for this comment. In review of the variables related to self-reported comorbid medical conditions, we found that there was not sufficient information regarding severity of illness, or definition of illness or recency of illness (apart from cancer and heart conditions) to reliably understand the influence of these variables on the outcomes of interest. We chose to include behavioral health conditions in the models given our specific interest in behavioral health related ED visits.

**Comment 6:** I deeply appreciate the sentiment of the sentence beginning line 137 on page 7 but I believe that the phrase "criminal tendencies" is not scientific and could be removed; consider naming structural racism as the underlying mechanism at play (citation if you need it : <https://www.nejm.org/doi/10.1056/NEJMms2025396>)

**Response**: Thank you for this comment. We have amended the text as suggested and as noted below.

- Page 11 Line182: *Race and ethnicity were chosen to be included in the model, not to imply any biological justification but as it is known that mass incarceration in the United States disproportionally impacts Black Americans, especially Black men, ^60^ and is likely due, in part, to racism at all levels including structural.^58,59^*

**Comment 7:** it does not appear the authors applied the necessary survey weights to analyze this data - see NSDUH codebook for guidance. If it was in fact done, it should be described in the methods

**Response:** Complex survey weighting as recommended by NSDUH was used. We have included this in the methods and updated the tables to make this explicit.

- Page 12 Line 211: We applied complex survey weighting as recommended by the NSDUH Codebook in all analyses.^53^

**Comment 8:** analysis plan for table 1 is not presented

**Response**: Thank you for this comment. We have amended the text as suggested.

- Page 12 Line 201: *We first performed descriptive analysis (count, proportion) of demographic characteristics and the exposures of interest (lifetime CLI and past year CLI) in the total population and by ED utilization in the prior 12 months.*

**Comment 9:** in general, the methods and results are not clearly described. I would recommend looking at each table, describing the methods for that table first, then the second table, then the third, etc.... Then do the same for the results.

**Response**: Thank you for this comment. We have amended the text as suggested and included markers for which tables correspond to the reported methods.

Results
**Comment 10** : there is no statistical analysis applied to the results in table 1 (ie p-values)

**Response**: We did not perform statistical tests in Table 1 and rather report purely descriptive results. Table 1 columns are separated by ED visit characteristics and are not mutually exclusive.

**Comment 11:** this is touched on above, but the presentation of the results needs to be more explicit about what is there. table 2 does not appear to be described in the methods or the results

**Response**: Thank you for this comment. We have amended the methods for clarity and comprehensiveness.

- Page 12 Line 201: *We first performed descriptive analysis (count, proportion) of demographic characteristics and the exposures of interest (lifetime CLI and past year CLI) in the total population and by ED utilization in the prior 12 months (Table 1). We then performed bivariate analysis using chi-squared tests of categorical variables to assess the relationship between lifetime CLI and past-year ED utilization and demographic characteristics. This was also done for past-year CLI (Table 2).*

Discussion
**Comment 12:** I would recommend that the authors focus their discussion specifically on exploring conversation tied directly to the findings.

**Response**: Thank you for this comment. We have significantly updated the discussion section to include focused and actionable recommendations tied to our findings and as noted above in response to other reviewers’ comments.

Thank you for taking the time to review and suggest improvements to this manuscript. We look forward to your feedback and appreciate opportunities to continue to revise as needed.

Sincerely,

Vidya Eswaran, MD MAS

Washington University School of Medicine

660 South Euclid Avenue, Box 8072,St. Louis, Missouri 63110

Phone: 314-362-4126 | [vidya@wustl.edu](mailto:vidya@wustl.edu)
