# Supplementary material for: Prevalence of criminal legal involvement among emergency department patients: Insights from the National Survey on Drug Use and Health 2021-2023
Source: PLoS One. 2026 Jul 8;21(7):e0351233. doi: 10.1371/journal.pone.0351233 (PMC13345442; doi:10.1371/journal.pone.0351233)
Supplement: Attachment — Submitted filename: NSDUH ED Arrest PLOSOne Response to Reviewers2.docx [file pone.0351233.s006.docx]

May 5, 2026

PLOS One

Ref: PONE-D-25-43064

Title: Prevalence of Criminal Legal Involvement Among Emergency Department Patients: Insights from the National Survey on Drug Use and Health 2021-2023

Authors: Vidya Eswaran, MD, Jun-Hong Chen PhD, Utsha G. Khatri, MD, Phillip L Marotta, PhD, Michael G Vaughn, PhD

Andrea K. Knittel, MD, PhD
Academic Editor, *PLOS One* Editorial Office

Dear Dr. Knittel,

We appreciate the insightful comments and suggestions provided by you and the review team for our manuscript, “Prevalence of Criminal Legal-Involvement Among Emergency Department Patients: Insights from the 2023 National Survey on Drug Use and Health”. Our team has carefully reviewed these insights and has revised our work accordingly. We also have highlighted relevant changes in the manuscript.

**Reviewer #1**: I want to congratulate the authors on a much improved manuscript and acknowledge how thoroughly and seriously they addressed the comments received in the prior round of review. I have only very minor comments to offer at this stage, the most substantial of the bunch refer to the methods section and should be very quick to resolve.

**Response:** Thank you for your comments and for your close review of the updated manuscript. We have addressed your comments as noted below.

Introduction

**Comment 1**: The abbreviation "ED" is not defined at first use

**Response:** This has been corrected on line 69.

**Comment 2**: The authors write "5x" rather than "five times"

**Response:** This has been corrected on line 73.

**Comment 3**: Typically, numerals less than 10 are spelled out (i.e. it should be "six months" rather than "6 months"). I will defer to the journal's style guide on this one

**Response:** This has been corrected throughout the manuscript.

**Comment 4**: Typically, U.S. is only used as an adjective (i.e. U.S. residents) and United States is only used as a noun (i.e. in the United States)

**Response:** This has been corrected throughout the manuscript.

**Comment 5**: There appears to be an extra carriage return on line 92

**Response:** This has been corrected.

METHODS

**Comment 6**: The mention of sampling limitations in the first paragraph is good, but the authors should further clarify how this is likely affecting the results. Right now, that impact on results is insinuated but not stated explicitly.

**Response:** Thank you for this comment. We have added the following to the methods section, line 161: “It is thus possible that our results may understate the prevalence of CLI in this population.”

**Comment 7**: It does not appear as though the authors took steps to control for multiple comparison in their chi-squared analyses (listed in Table 2). This may be de facto handled by the fact that the table only indicates when p<0.001, but at least some mention of the authors awareness of the risk of false discovery and source of confidence in their presented data should be made. Even as a footnote to the table would be fine.

**Response:** Thank you for this comment. We have completed a simple chi square test applied with a Bonferroni correction as well as complex sample weighting, and found the same significance patterns (i.e., significance is still significance).

We have added the following footnote to the bottom of Table 2: “To address potential false discovery, a Bonferroni correction was applied, and statistical significance was assessed using the adjusted significance threshold. The results remained unchanged.

RESULTS

**Comment 8**: at least one of the "p"s in table 2 is capitalized.

**Response:** This has been corrected in Table 2.

Thank you for taking the time to review and suggest improvements to this manuscript. We look forward to your feedback and appreciate opportunities to continue to revise as needed.

Sincerely,

Vidya Eswaran, MD MAS

Washington University School of Medicine

660 South Euclid Avenue, Box 8072,St. Louis, Missouri 63110

Phone: 314-362-4126 | [vidya@wustl.edu](mailto:vidya@wustl.edu)
