# Supplementary material for: Prevalence of criminal legal involvement among emergency department patients: Insights from the National Survey on Drug Use and Health 2021-2023
Source: PLoS One. 2026 Jul 8;21(7):e0351233. doi: 10.1371/journal.pone.0351233 (PMC13345442; doi:10.1371/journal.pone.0351233)
Supplement: Peer review history

# Peer review: Decision Letter 0

**DOI:** 10.1371/journal.pone.0351233.r001

**Authors:** Andrea K. Knittel

15 Dec 2025

PONE-D-25-43064

Prevalence of Criminal Legal-Involvement Among Emergency Department Patients: Insights from the 2023 National Survey on Drug Use and Health

PLOS One

Dear Dr. Eswaran,

Thank you for submitting your manuscript to PLOS ONE. After careful consideration, we feel that it has merit but does not fully meet PLOS ONE’s publication criteria as it currently stands. Therefore, we invite you to submit a revised version of the manuscript that addresses the points raised during the review process.

Please submit your revised manuscript by Jan 29 2026 11:59PM. If you will need more time than this to complete your revisions, please reply to this message or contact the journal office at plosone@plos.org. When you're ready to submit your revision, log on to https://www.editorialmanager.com/pone/ and select the 'Submissions Needing Revision' folder to locate your manuscript file.

Please include the following items when submitting your revised manuscript:

- A rebuttal letter that responds to each point raised by the academic editor and reviewer(s). You should upload this letter as a separate file labeled 'Response to Reviewers'.
- A marked-up copy of your manuscript that highlights changes made to the original version. You should upload this as a separate file labeled 'Revised Manuscript with Track Changes'.
- An unmarked version of your revised paper without tracked changes. You should upload this as a separate file labeled 'Manuscript'.

If you would like to make changes to your financial disclosure, please include your updated statement in your cover letter. Guidelines for resubmitting your figure files are available below the reviewer comments at the end of this letter.

If applicable, we recommend that you deposit your laboratory protocols in protocols.io to enhance the reproducibility of your results. Protocols.io assigns your protocol its own identifier (DOI) so that it can be cited independently in the future. For instructions see: https://journals.plos.org/plosone/s/submission-guidelines#loc-laboratory-protocols. Additionally, PLOS ONE offers an option for publishing peer-reviewed Lab Protocol articles, which describe protocols hosted on protocols.io. Read more information on sharing protocols at https://plos.org/protocols?utm_medium=editorial-email&utm_source=authorletters&utm_campaign=protocols.

We look forward to receiving your revised manuscript.

Kind regards,

Andrea K. Knittel

Academic Editor

PLOS One

Journal Requirements:

When submitting your revision, we need you to address these additional requirements.

1. Please ensure that your manuscript meets PLOS ONE's style requirements, including those for file naming. The PLOS ONE style templates can be found at

https://journals.plos.org/plosone/s/file?id=wjVg/PLOSOne_formatting_sample_main_body.pdf and

https://journals.plos.org/plosone/s/file?id=ba62/PLOSOne_formatting_sample_title_authors_affiliations.pdf

2. Please note that your Data Availability Statement is currently missing the direct link to access each database. If your manuscript is accepted for publication, you will be asked to provide these details on a very short timeline. We therefore suggest that you provide this information now, though we will not hold up the peer review process if you are unable.

3. Please include captions for your Supporting Information files at the end of your manuscript, and update any in-text citations to match accordingly. Please see our Supporting Information guidelines for more information: http://journals.plos.org/plosone/s/supporting-information.

4. If the reviewer comments include a recommendation to cite specific previously published works, please review and evaluate these publications to determine whether they are relevant and should be cited. There is no requirement to cite these works unless the editor has indicated otherwise.

**Additional Editor Comments:**

There are multiple substantive concerns about the framing and the analysis that the reviewers have raised. Although the PLOS ONE publication criteria do not require that the analysis is novel, they do require that it is clearly presented and that the conclusions are wholly founded in the data and analysis. To achieve this, I recommend careful consideration of each of the reviewer comments. Without addressing all of them, this manuscript is unlikely to meet the PLOS ONE publication criteria.

[Note: HTML markup is below. Please do not edit.]

Reviewers' comments:

Reviewer's Responses to Questions

**Comments to the Author**

1. Is the manuscript technically sound, and do the data support the conclusions?

The manuscript must describe a technically sound piece of scientific research with data that supports the conclusions. Experiments must have been conducted rigorously, with appropriate controls, replication, and sample sizes. The conclusions must be drawn appropriately based on the data presented.

Reviewer #1: Partly

Reviewer #2: Yes

Reviewer #3: Yes

Reviewer #4: No

**********

2. Has the statistical analysis been performed appropriately and rigorously?

Reviewer #1: I Don't Know

Reviewer #2: Yes

Reviewer #3: Yes

Reviewer #4: No

**********

3. Have the authors made all data underlying the findings in their manuscript fully available?

The PLOS Data policy requires authors to make all data underlying the findings described in their manuscript fully available without restriction, with rare exception (please refer to the Data Availability Statement in the manuscript PDF file). The data should be provided as part of the manuscript or its supporting information, or deposited to a public repository. For example, in addition to summary statistics, the data points behind means, medians and variance measures should be available. If there are restrictions on publicly sharing data—e.g. participant privacy or use of data from a third party—those must be specified.

Reviewer #1: Yes

Reviewer #2: Yes

Reviewer #3: Yes

Reviewer #4: Yes

**********

4. Is the manuscript presented in an intelligible fashion and written in standard English?

PLOS ONE does not copyedit accepted manuscripts, so the language in submitted articles must be clear, correct, and unambiguous. Any typographical or grammatical errors should be corrected at revision, so please note any specific errors here.

Reviewer #1: Yes

Reviewer #2: Yes

Reviewer #3: Yes

Reviewer #4: No

**********

5. Review Comments to the Author

Please use the space provided to explain your answers to the questions above. You may also include additional comments for the author, including concerns about dual publication, research ethics, or publication ethics. (Please upload your review as an attachment if it exceeds 20,000 characters)

Reviewer #1: This paper presents an analysis of the relationship between emergency department utilization and recent or lifetime criminal legal involvement using data from the 2023 national survey on drug use and health. The paper presents an interesting analysis that certainly has interest and utility in our current social and policy environment. However, the analysis lacks a certain degree of clarity in its methodology—specifically related to matters of sampling and measurement—and its significance or impact for practitioners and policymakers in general.

To begin with the significance or impact of this analysis, the utility of the author's findings to policy or practice is not sufficiently articulated in the manuscript. Throughout the abstract as well as the manuscript itself (including on page 4 in the introduction, page 5 in the introduction, and page 15 and 16 in the discussion), the authors repeatedly mention the need for emergency medicine providers to better understand and address or to better identify and treat the specific needs of people presenting to an emergency department with a history of criminal legal involvement. However, what those specific patient needs or provider actions that might be is never clearly articulated in the draft, and I am struggling to imagine what particular, individualized intervention any healthcare practitioner could take that would target an underlying structural driver of the surface level biological or psychological concern they are there to treat. In other words, how should a healthcare provider treat a person presenting to the emergency department with a substance use related issue any differently based on their history (or lack thereof) of criminal legal involvement?

I don't mean to throw the baby out with the bathwater and say that healthcare providers have nothing to offer in terms of structural supports to the people that they serve. Nevertheless, the authors failed to clarify how the needs of patient presenting to an emergency department with a mental illness or substance use related concern would be fundamentally different according to that individual's current or prior criminal legal involvement. The practical details of this distinction is of the utmost importance to the argument that the authors appear to be trying to make. Yet it is glaringly absent from the paper. Only in the discussion section do the authors begin to theorize how criminal legal involvement of any kind might have a direct impact on the likelihood of someone's presentation to the emergency department, identifying lasting trauma from criminal legal involvement as a driver of behavioral health concerns or lack of access to basic life needs and financial resources as a driver of emergency department utilization. (Importantly, the authors don't explicitly identify these as plausible mechanisms explaining their findings, but they absolutely should.) Yet those plausible mechanisms don't to address at all how people with past criminal legal involvement might somehow present differently to an emergency department or how the needs they present with might be fundamentally different or how they should be addressed in some specific, targeted way. In other words, it's not at all clear how healthcare providers are supposed to treat people with criminal legal involvement differently or understand their problems through some different framework.

On page 16 the authors do make a couple of suggestions about what that could look like in a practical sense, but those suggestions are limited to a brief mention of connecting patients with reentry focused resources, which—I feel quite strongly should be noted is not a healthcare provider's job; That is the job of jails and prisons and existing state-run reentry services—and building collaborations and supports across hospital departments, which would be a benefit to all patients presenting with behavioral health issues not just those with criminal legal involvement. So, in the end, it's not at all clear what practitioners or policymakers are supposed to do with the information provided in this manuscript especially insofar as the authors are focusing on individual level health care practices more so than structural changes or population level policy changes that might begin to address the problematic relationships their analysis identifies.

With regard to measurement and method, I understand that the authors are limited to the indicators that were already included in the national survey on drug use and health. However, it seems to me that the problems with using a measurement like arrest and booking as an indicator for criminal legal involvement should be obvious and openly discussed both in the plan for analysis and in the limitations section of the paper. What arrest and booking is able to measure is, in a way, NEW episodes of criminal legal involvement. Or perhaps even ongoing episodes of criminal legal involvement. I'm thinking here of someone who has been on community supervision for 18 months after release from a period of incarceration has not been arrested or booked during that time period. That person absolutely has been subject to criminal legal involvement during the past 12 months but that would not be identified by the measures used in the study. Thus one major problem with this measurement is the fact that people who are under community supervision or some other degree of criminal legal involvement that has not resulted in an arrest on a booking in the past 12 months cannot be identified and assigned to the treatment group as appropriate.

Further, the sampling limitations that are inherent in the national survey on drug use and health methodology need to be articulated in the methods section, not just the limitations section and the impact of those sampling challenges on the results that the authors have produced and need to be discussed much more thoroughly and clearly in the paper. Lots of other scholars have addressed the deeply ironic sampling challenges with this survey, so the authors are well within their rights to cite out to other works that have already produced this kind of analysis of the surveys sampling structure and the problems it injects into its data. But it has to be brought up explicitly ahead of the analysis in paper.

A few minor comments follow:

1) In the opening lines of the paper, the authors make reference to “Western countries.” This term can be a bit problematic and it's usually best to avoid using it whenever possible. If the authors simply mean that the United States has incarcerated more people than other countries in Europe and North America, say that. If the author is mean that the United States has the highest incarceration rate of countries with GDP above a certain amount, say that. It's always preferable to just say what you mean instead of glossing it with squishy and problematic terms like “western.”

2) There seems to be a citation missing at the end of the second sentence, ending in the phrase “drug related crime.”

3) The authors use the term prisoners at the end of the first paragraph. This term is not preferred.

4) On page 4 the authors refer to other sources of care that are decreasing in the United States. It would be helpful to state explicitly what those are.

5) The authors do a good job in the measures section of explaining what the term booked means, but a brief explanation of how that connects to other forms of criminal legal involvement (as in being booked is the gateway to other forms of criminal legal involvement like prosecution, drug court, community service, probation, etc.) would be helpful.

6) On page 14 the authors state that their findings suggest associations between criminal legal involvement and emergency department use persist over time. In this context, the term persists suggests a temporal model of causality between criminal legal involvement and emergency department utilization that is not supported by this study and seems plausible but unlikely given the other bodies of evidence that exist, many of which the authors are already referring to. Put another way, many socioeconomic factors themselves are predictive of both criminal legal involvement and emergency department utilization, so if the authors want to suggest that an effect of criminal legal involvement persists over time they need to establish that something is an effect of criminal legal involvement. Whether through their own data or through reference to other literature, they have not done so here.

7) The study mentioned in the discussion that shows very low levels of criminal legal involvement among emergency department patients via an analysis of electronic health records seems important context for the introduction.

Reviewer #2: This is an interesting manuscript on an important topic which is the impact of criminal justice involvement in use of emergency departments. There are a few areas where the grammar needs to be reviewed and some spelling errors (see line 135 "non-Hispanic Back should be "Black").

The authors effectively highlight the intersection of incarceration, substance use, mental health, and healthcare access, particularly in ED settings. The justification for focusing on lifetime CLI rather than only recent involvement is persuasive and adds depth to the study's relevance.

The dataset is nationally representative but has its drawbacks which are discussed in the limitations section.

The methodology is robust, employing descriptive statistics, logistic regression, and propensity score weighting to account for confounding variables. The paper would benefit from a little more discussion of the use of both logistic regression and propensity score analysis, and how they complement each other and why both are needed, especially with respect to Table 4.

While it is not a novel finding that formerly incarcerated individuals access the ED for healthcare, and that these are important social determinants of health, the manuscript adds incrementally to our knowledge of the impact of incarceration on healthcare-seeking behavior.

The authors advocate for screening and tailored interventions in EDs, which is a logical extension of their findings. However, as the authors note, that could be a heavy lift as most EDs do not collect that information and the authors themselves state that it is not clear or established how data on previous incarceration would be collected in that setting.

Finally, the paper could use additional discussion on whether findings from these data are truly generalizable.

Reviewer #3: Authors: This study uses a retrospective cohort design from publicly available nationally representative sample to look at criminal legal system involvement among those with a past year emergency department visit. This is a novel question that has not been previously undertaken to my knowledge. It can be an important question, given that health disparities are well described amongst people with justice involvement, compared with the general population, and identifying these individuals may provide an opportunity for targeted health system interventions such as linkage to peers, medical legal partnerships, rapid housing and targeted primary care programs.

This manuscript will benefit from more clearly laying out the current state of knowledge on this topic and what specifically this study adds, as well as the specific research question. I do believe that with moderate edits, this manuscript can contribute meaningfully to the literature on healthcare delivery and quality of community care delivery for previously incarcerated people, especially where they most often access care- in the emergency department.

Title: Suggest remove hyphen from title.

Abstract: Not clear to the average reader why this is a problem. No mention of health risks, so why should EDs care if there is past CLI? Opportunity to more clearly state the problem in the intro. Advise reorder the results for ED visits among CLI line 45-46 in abstract so that consistent with reporting order in the sentences prior. It is otherwise more confusing for the reader.

Introduction:

As currently written it is challenging to follow and could be tightened. The authors present a broad overall epidemiology of incarceration in the US, of substance use disorder treatment for those with CLI, then social determinants, and then to the role of the ED in the life of people with CLI. I suggest really tightening and getting to the health risks right away and then the way this population uses the ED and why identifying CLI might be helpful in the ED. Why is it any more helpful that just identifying the SUD or the mental health condition? These patients are also often unstably housed and with many social determinant needs. Again, why is this helpful? What might the ED do different for this population than any other population with SUD or mental health conditions? Line 78-79 mentions this vaguely, but being more specific would be helpful in setting up the value of this line of inquiry. Additionally would be most helpful to summarize the literature on ED use for this population and what the gaps really are. As currently written this introduction does not present in detail what are the past findings on ED use for this populations and what are the limitations of the current body of research. This would set up for a stronger final paragraph where the authors can more clearly and specifically define the research question. For example, the first author has previously published work that established that frequent ED visits are associated with subsequent jail incarceration and that the ED is a place for intervention to prevent incarceration- how does this study build off of that work? Is the question what is spelled out in second sentence of the abstract? Is this really what you sought to assess?: “We sought to assess lifetime and past year CLI among those reporting an ED visit for any reason, for substance use and for mental health.” Having read through the manuscript this does not appear to be the primary question, your methods and results sections focus much more on differential use of the ED for people with and without CLI, so is the question how people with CLI use the ED or is the question what percent of people going to an ED have CLI? If your question is as stated, then Table 1 makes sense, but then why Table 2 and related analysis? What does this study add that data linkage studies that have linked jail and ED records have not addressed (see Easter et al, Journal of Correctional Healthcare, 2023; Humphreys, JUH, 2017) and what does this add beyond the work of Joseph Frank et al in JGIM that you cited but really didn’t expand upon- they did a similar analysis, why is this study needed and different?

Methods:

Why did the authors not include the question about probation or parole in defining CLI? Past studies have. If there was a strong rationale, would explicate here. If not including this, why not call this variable what it is, which is prior incarceration, not the broader conceptualization of criminal legal system involvement which typically includes incarceration and community supervision through probation and parole.

Also, why the decision to use one year of NSDUH data instead of combining multiple years to increase sample of people with recent CLI and different ED use?

Why did the authors feel that propensity score matching was necessary when past studies using NSDUH with this population have not employed this approach, especially given that there are not that many other variables to control for between CLI and non-CLI? It would be add to your methods if you describe the need for this methodology more and what deficits it responds to in prior studies using NSDUH.

Discussion: Overall the discussion feels more compelling than the introduction and gets the reader to make sense of the results. I think one thing that could be teased out more is what health systems would even do about any prior lifetime CLI exposure? The argument to identify recent release or recent CLI is certainly more supported by the literature and feels more actionable.

281-283- unclear what this sentence means

296-298- in fact, data from NSDUH suggest that the increase in Medicaid post ACA expansion was not associated with increased engagement among people with CLI. (Howell et al, Psych Services, 2023), so this would be helpful to add here and contextualize your findings based on these past findings.

Table 2: N should be in first row for CLI vs no CLI.

Reviewer #4: 1. Introduction

- this section could focus on statements which directly support the scientific question at hand. The second and third paragraphs do not seem to fit where they have been placed.

- The problems created by the MIEP go much further than access to health insurance - it creates a siloed carceral health system and prevents funding for transitional services. This is a nice review of the matter, it would be worthwhile to update the sentence begining line 71 on page 2: https://pmc.ncbi.nlm.nih.gov/articles/PMC11281997/

- the first paragraph on page 5, is not very scholarly. While there are many gaps in knowledge about CLI and ED utliization, there is a not a dearth of studies on the matter either. Moreover, many of the studies that you cite use the same datasource as this paper, so more clearly identifying the precise gap in knowledge would strengthen the scientific rigor of this introduction.

Methods

- Is there any reason that the authors did not combine this data set with 2022 or 2021? The N for several outcome groups is very small, limiting interpretation of these results. Datasets can be combined per NSDUH recommended procedures (though I believe 2020 should not be combined so you may be limited is 2021-2023.

- Given the outcome is healthcare utlization, and variables on other comorbid health conditions is available in the dataset, it seems appropriate to control for these as well.

- I deeply appreciate the sentiment of the sentence beginning line 137 on page 7 but I believe that the phrase "criminal tendencies" is not scientific and could be removed; consider naming structural racism as the underlying mechanism at play (citation if you need it : https://www.nejm.org/doi/10.1056/NEJMms2025396)

- it does not appear the authors applied the necessary survey weights to analyze this data - see NSDUH codebook for guideance. If it was in fact done, it should be described in the methods

- analysis plan for table 1 is not presented

- in general, the methods and results are not clearly described. I would reccomend looking at each table, describing the methods for that table first, then the second table, then the third, etc.... Then do the same for the results.

Results

- there is no statistical analysis applied to the results in table 1 (ie p-values)

- this is touched on above, but the presentation of the results needs to be more explicit about what is there. table 2 does not appear to be described in the methods or the results

Discussion

- I would recommend that the authors focus their discussion specifically on exploring conversation tied directly to the findings.

**********

6. PLOS authors have the option to publish the peer review history of their article (what does this mean?). If published, this will include your full peer review and any attached files.

If you choose “no”, your identity will remain anonymous but your review may still be made public.

**Do you want your identity to be public for this peer review?**   For information about this choice, including consent withdrawal, please see our Privacy Policy.

Reviewer #1: No

Reviewer #2: **Yes:**  Grace Reynolds-Fisher

Reviewer #3: No

Reviewer #4: No

**********

[NOTE: If reviewer comments were submitted as an attachment file, they will be attached to this email and accessible via the submission site. Please log into your account, locate the manuscript record, and check for the action link "View Attachments". If this link does not appear, there are no attachment files.]

To ensure your figures meet our technical requirements, please review our figure guidelines: https://journals.plos.org/plosone/s/figures

You may also use PLOS’s free figure tool, NAAS, to help you prepare publication quality figures: https://journals.plos.org/plosone/s/figures#loc-tools-for-figure-preparation.

NAAS will assess whether your figures meet our technical requirements by comparing each figure against our figure specifications.

# Author comment: Author response to Decision Letter 1

**DOI:** 10.1371/journal.pone.0351233.r002

23 Mar 2026

March 16, 2026

PLOS One

Ref: PONE-D-25-43064

Title: Prevalence of Criminal Legal Involvement Among Emergency Department Patients: Insights from the National Survey on Drug Use and Health 2021-2023

Authors: Vidya Eswaran, MD, Jun-Hong Chen PhD, Utsha G. Khatri, MD, Phillip L Marotta, PhD, Michael G Vaughn, PhD

Andrea K. Knittel, MD, PhD

Academic Editor, PLOS One Editorial Office

Dear Dr. Knittel,

We appreciate the insightful comments and suggestions provided by you and the review team for our manuscript, “Prevalence of Criminal Legal-Involvement Among Emergency Department Patients: Insights from the 2023 National Survey on Drug Use and Health”. Our team has carefully reviewed these insights and has revised our work accordingly. We also have highlighted relevant changes in the manuscript.

Reviewer #1: This paper presents an analysis of the relationship between emergency department utilization and recent or lifetime criminal legal involvement using data from the 2023 national survey on drug use and health. The paper presents an interesting analysis that certainly has interest and utility in our current social and policy environment. However, the analysis lacks a certain degree of clarity in its methodology—specifically related to matters of sampling and measurement—and its significance or impact for practitioners and policymakers in general.

• Response: Thank you for this comment and for allowing us the opportunity to make edits to address concerns related to clarity in methodology and significance to practitioners and policy makers.

Comment 1: To begin with the significance or impact of this analysis, the utility of the author's findings to policy or practice is not sufficiently articulated in the manuscript. Throughout the abstract as well as the manuscript itself (including on page 4 in the introduction, page 5 in the introduction, and page 15 and 16 in the discussion), the authors repeatedly mention the need for emergency medicine providers to better understand and address or to better identify and treat the specific needs of people presenting to an emergency department with a history of criminal legal involvement. However, what those specific patient needs or provider actions that might be is never clearly articulated in the draft, and I am struggling to imagine what particular, individualized intervention any healthcare practitioner could take that would target an underlying structural driver of the surface level biological or psychological concern they are there to treat. In other words, how should a healthcare provider treat a person presenting to the emergency department with a substance use related issue any differently based on their history (or lack thereof) of criminal legal involvement?

I don't mean to throw the baby out with the bathwater and say that healthcare providers have nothing to offer in terms of structural supports to the people that they serve. Nevertheless, the authors failed to clarify how the needs of patient presenting to an emergency department with a mental illness or substance use related concern would be fundamentally different according to that individual's current or prior criminal legal involvement. The practical details of this distinction is of the utmost importance to the argument that the authors appear to be trying to make. Yet it is glaringly absent from the paper. Only in the discussion section do the authors begin to theorize how criminal legal involvement of any kind might have a direct impact on the likelihood of someone's presentation to the emergency department, identifying lasting trauma from criminal legal involvement as a driver of behavioral health concerns or lack of access to basic life needs and financial resources as a driver of emergency department utilization. (Importantly, the authors don't explicitly identify these as plausible mechanisms explaining their findings, but they absolutely should.) Yet those plausible mechanisms don't to address at all how people with past criminal legal involvement might somehow present differently to an emergency department or how the needs they present with might be fundamentally different or how they should be addressed in some specific, targeted way. In other words, it's not at all clear how healthcare providers are supposed to treat people with criminal legal involvement differently or understand their problems through some different framework.

On page 16 the authors do make a couple of suggestions about what that could look like in a practical sense, but those suggestions are limited to a brief mention of connecting patients with reentry focused resources, which—I feel quite strongly should be noted is not a healthcare provider's job; That is the job of jails and prisons and existing state-run reentry services—and building collaborations and supports across hospital departments, which would be a benefit to all patients presenting with behavioral health issues not just those with criminal legal involvement. So, in the end, it's not at all clear what practitioners or policymakers are supposed to do with the information provided in this manuscript especially insofar as the authors are focusing on individual level health care practices more so than structural changes or population level policy changes that might begin to address the problematic relationships their analysis identifies.

Response: Thank you for this insightful comment. We agree with the reviewer that further development of next steps and the clinical impact of these findings is needed. At the individual-level, we believe that many ED clinicians are likely unaware that CLSI impacts such many ED patients. CLSI is a known risk factor for short and long term health outcomes and is associated with premature mortality. It is important that clinicians are educated on these risks and to consider them when developing treatment plans and referral prioritization. Further, while in recent years focused training on the provision of trauma-informed care has become more common, we believe it vitally important for clinicians to be aware of the harms caused by incarceration and how it may impact their interaction with patients. We have included more specific examples of ED based interventions and included ED social workers, case managers and hospital and health care leaders as important stakeholders when planning interventions to address the needs of patients with CLSI. Selected text has been included below highlighting these changes.

• Page 7 Line 111: Understanding which patients are more likely to rely on ED care, and why, may help inform more tailored and equitable ED-based interventions.

Although patients with CLI experience higher risks for adverse health outcomes, it remains unclear whether and how knowledge of CLI adds meaningful information beyond the identification of SUD, psychiatric disease, or other social needs commonly encountered among ED patients. While social determinant screening is becoming more common in health care settings 31–33, screening for CLI is rare, and its potential utility in ED care has not been well defined. The existing literature on ED utilization among people with CLI focuses on cohorts with known and recent incarceration, often identified through administrative data linkages. 18,27,34 Less is known about the prevalence of lifetime CLI among the broader ED population, or whether individuals with a history of CLI, regardless of how recent, display different patterns of ED utilization. Without such data, designing targeted interventions and understanding the role ED settings can play in addressing substance use and mental health needs in this population remains challenging

• Page 19 Line 349: There is growing understanding of the importance of competency in the provision of trauma informed care among ED clinicians.55 Given the significant trauma experienced by many while incarcerated,56 that mistrust of health care systems and providers persist after release from carceral facilities, and that many people who were formally incarcerated report bias from healthcare providers,57,58 knowing that 20% of ED patients have experienced incarceration can be helpful in motivating clinicians to be prepared to deliver trauma-informed care. Practically, this could include lectures to medical students and trainees about mass incarceration and the short- and long-term health impacts, including personal or familial incarceration in lectures related to social determinants of health, inviting individuals with lived experience of incarceration to share their experience in lectures or small groups, and frequent training and skills building in best practices related to the provision of trauma informed care.

Incarceration has been associated with increased risk of adverse medical and behavioral health outcomes and premature mortality.59–62 It is important that ED clinicians are aware of these risks and consider them when developing treatment plans and prioritizing patient referrals for outpatient care. Beyond, individual level care, awareness of high prevalence of CLI among ED patients is important for health systems when considering interventions and resource development. EDs have long been centers of implementation for public health screening and intervention.63–65 Multiple ED-based programs exist to address basic needs such as food and housing insecurity, for example.66–68 Universal screening of ED patients for HIV have improved rates of diagnoses and linkage to care.69 Given proposed and recent changes to Medicaid policy, more individuals may lose access to Medicaid and thus rely on EDs for care beyond their acute, emergent needs.70 Awareness of CLI among a large portion of ED patients should inspire careful consideration by ED and hospital leadership to consider how ED staff and health care systems can best support this population. ED-based social workers and case managers may be able to connect patients with re-entry focused resources within their community to provide continued support beyond the hospital walls. Medical-Legal partnerships71,72 could be developed to bring comprehensive social services to patients being seen in the ED. Collaboration between addiction medicine, mental health clinics and EDs could include warm handoffs between clinical sites, and assistance with implementation of protocols to facilitate the provision of medications for substance use disorders in the ED to patients who may otherwise have limited access to outpatient healthcare, especially given known disparities in access to standard of care treatments among those with CLI. The ED could be a meaningful point of intervention and partner to prevent adverse health outcomes, and potentially recidivism and re-incarceration as well.

Comment 2: With regard to measurement and method, I understand that the authors are limited to the indicators that were already included in the national survey on drug use and health. However, it seems to me that the problems with using a measurement like arrest and booking as an indicator for criminal legal involvement should be obvious and openly discussed both in the plan for analysis and in the limitations section of the paper. What arrest and booking is able to measure is, in a way, NEW episodes of criminal legal involvement. Or perhaps even ongoing episodes of criminal legal involvement. I'm thinking here of someone who has been on community supervision for 18 months after release from a period of incarceration has not been arrested or booked during that time period. That person absolutely has been subject to criminal legal involvement during the past 12 months but that would not be identified by the measures used in the study. Thus one major problem with this measurement is the fact that people who are under community supervision or some other degree of criminal legal involvement that has not resulted in an arrest on a booking in the past 12 months cannot be identified and assigned to the treatment group as appropriate.

Response: Thank you for this comment. We agree that the use of arrest or booking as a marker for past-year CLSI is limited for the reasons you have mentioned. We include lifetime CLSI as a key exposure in our study partly for this reason. We have added language to the text to further highlight this limitation.

• Page 10 Line 164: Past year CLI was defined as an answer of greater than 0 to the question “Not counting minor traffic violations, how many times during the past 12 months have you been arrested and booked for breaking a law?” and serves as a marker of new involvement with the criminal legal system.

• Page 23 Line 406: Fourth, the use of ‘arrest and booking’ to define CLI, especially in the past-year, limits CLI to new episodes of criminal legal involvement and fails to capture those under community supervision who, while still ‘involved’ with the system, may not have been arrested or booked during the prior twelve months. The use of lifetime CLI is intended to more broadly include this population.

Comment 3: Further, the sampling limitations that are inherent in the national survey on drug use and health methodology need to be articulated in the methods section, not just the limitations section and the impact of those sampling challenges on the results that the authors have produced and need to be discussed much more thoroughly and clearly in the paper. Lots of other scholars have addressed the deeply ironic sampling challenges with this survey, so the authors are well within their rights to cite out to other works that have already produced this kind of analysis of the surveys sampling structure and the problems it injects into its data. But it has to be brought up explicitly ahead of the analysis in paper.

Response: Thank you for this comment. We have included the following text in the Methods section of the manuscript:

• Page 9 Line 148: Given the sampling strategy of NSDUH it is important to contextualize the study understanding that housing insecure and currently incarcerated or otherwise institutionalized individuals are not included, especially given that both those these social risks are associated both with increased risk of incarceration and ED use.27,36,37

Comment 4: In the opening lines of the paper, the authors make reference to “Western countries.” This term can be a bit problematic and it's usually best to avoid using it whenever possible. If the authors simply mean that the United States has incarcerated more people than other countries in Europe and North America, say that. If the author is mean that the United States has the highest incarceration rate of countries with GDP above a certain amount, say that. It's always preferable to just say what you mean instead of glossing it with squishy and problematic terms like “western.”

Response: Thank you for this comment. We have amended this statement as noted below.

• Page 4 Line 55: The United States (US) incarcerates more of its citizens than any other high-income country, with incarceration rates exceeding those of peer nations

Comment 5: There seems to be a citation missing at the end of the second sentence, ending in the phrase “drug related crime.”

Response: Thank you for this comment. References have been added.

Comment 6: The authors use the term prisoners at the end of the first paragraph. This term is not preferred.

Response: Thank you for this comment. We have changed the text as noted below:

• Page 5 Line 79: It is estimated that 30-45% of those incarcerated in prison have at least one chronic medical condition or disease, 5,6 and many report current or prior infectious disease history, such as HIV or Hepatitis C.5

Comment 7: On page 4 the authors refer to other sources of care that are decreasing in the United States. It would be helpful to state explicitly what those are.

Response: Thank you for this comment. We have amended the text as noted below:

• Page 6 Line 105: As EDs increasingly become an important access point to identify and treat substance use disorders 26, and in an era within the US where access to other sources of care, such as inpatient psychiatric facilities and safety-net clinics, may be decreasing,27,28 their role in addressing health disparities related to CLI is gaining attention. 29,30

Comment 8: The authors do a good job in the

Attachment Submitted filename: NSDUH ED Arrest PLOSOne Response to Reviewers.docx

# Peer review: Decision Letter 1

**DOI:** 10.1371/journal.pone.0351233.r003

**Authors:** Andrea K. Knittel

3 May 2026

PONE-D-25-43064R1

Prevalence of Criminal Legal Involvement Among Emergency Department Patients: Insights from the National Survey on Drug Use and Health 2021-2023

PLOS One

Dear Dr. Eswaran,

Thank you for submitting your manuscript to PLOS ONE. This revision was extremely responsive to the reviewers comments and very nearly meets all of the PLOS ONE publication criteria. The reviewers did identify a few small changes that are needed to ensure that the study is clearly and accurately reported. Therefore, we invite you to submit a revised version of the manuscript that addresses the last few points raised during the review process.

Please submit your revised manuscript by Jun 17 2026 11:59PM. If you will need more time than this to complete your revisions, please reply to this message or contact the journal office at plosone@plos.org. When you're ready to submit your revision, log on to https://www.editorialmanager.com/pone/ and select the 'Submissions Needing Revision' folder to locate your manuscript file.

Please include the following items when submitting your revised manuscript:

- A letter that responds to each point raised by the academic editor and reviewer(s). You should upload this letter as a separate file labeled 'Response to Reviewers'.
- A marked-up copy of your manuscript that highlights changes made to the original version. You should upload this as a separate file labeled 'Revised Manuscript with Track Changes'.
- An unmarked version of your revised paper without tracked changes. You should upload this as a separate file labeled 'Manuscript'.

If you would like to make changes to your financial disclosure, please include your updated statement in your cover letter. Guidelines for resubmitting your figure files are available below the reviewer comments at the end of this letter.

If applicable, we recommend that you deposit your laboratory protocols in protocols.io to enhance the reproducibility of your results. Protocols.io assigns your protocol its own identifier (DOI) so that it can be cited independently in the future. For instructions see: https://journals.plos.org/plosone/s/submission-guidelines#loc-laboratory-protocols. Additionally, PLOS ONE offers an option for publishing peer-reviewed Lab Protocol articles, which describe protocols hosted on protocols.io. Read more information on sharing protocols at https://plos.org/protocols?utm_medium=editorial-email&utm_source=authorletters&utm_campaign=protocols.

As the corresponding author, your ORCID iD is verified in the submission system and will appear in the published article. PLOS supports the use of ORCID, and we encourage all coauthors to register for an ORCID iD and use it as well. Please encourage your coauthors to verify their ORCID iD within the submission system before final acceptance, as unverified ORCID iDs will not appear in the published article. *Only*   the individual author can complete the verification step; PLOS staff *cannot*   verify ORCID iDs on behalf of authors.

We look forward to receiving your revised manuscript.

Kind regards,

Andrea K. Knittel

Academic Editor

PLOS One

Journal Requirements:

1. If the reviewer comments include a recommendation to cite specific previously published works, please review and evaluate these publications to determine whether they are relevant and should be cited. There is no requirement to cite these works unless the editor has indicated otherwise.

2. Please review your reference list to ensure that it is complete and correct. If you have cited papers that have been retracted, please include the rationale for doing so in the manuscript text, or remove these references and replace them with relevant current references. Any changes to the reference list should be mentioned in the rebuttal letter that accompanies your revised manuscript. If you need to cite a retracted article, indicate the article’s retracted status in the References list and also include a citation and full reference for the retraction notice.

[Note: HTML markup is below. Please do not edit.]

Reviewers' comments:

Reviewer's Responses to Questions

**Comments to the Author**

1. If the authors have adequately addressed your comments raised in a previous round of review and you feel that this manuscript is now acceptable for publication, you may indicate that here to bypass the “Comments to the Author” section, enter your conflict of interest statement in the “Confidential to Editor” section, and submit your "Accept" recommendation.

Reviewer #1: All comments have been addressed

Reviewer #2: All comments have been addressed

Reviewer #4: All comments have been addressed

**********

2. Is the manuscript technically sound, and do the data support the conclusions?

The manuscript must describe a technically sound piece of scientific research with data that supports the conclusions. Experiments must have been conducted rigorously, with appropriate controls, replication, and sample sizes. The conclusions must be drawn appropriately based on the data presented.

Reviewer #1: Yes

Reviewer #2: Yes

Reviewer #4: Yes

**********

3. Has the statistical analysis been performed appropriately and rigorously?

Reviewer #1: Yes

Reviewer #2: Yes

Reviewer #4: Yes

**********

4. Have the authors made all data underlying the findings in their manuscript fully available?

The PLOS Data policy requires authors to make all data underlying the findings described in their manuscript fully available without restriction, with rare exception (please refer to the Data Availability Statement in the manuscript PDF file). The data should be provided as part of the manuscript or its supporting information, or deposited to a public repository. For example, in addition to summary statistics, the data points behind means, medians and variance measures should be available. If there are restrictions on publicly sharing data—e.g. participant privacy or use of data from a third party—those must be specified.

Reviewer #1: Yes

Reviewer #2: Yes

Reviewer #4: Yes

**********

5. Is the manuscript presented in an intelligible fashion and written in standard English?

PLOS ONE does not copyedit accepted manuscripts, so the language in submitted articles must be clear, correct, and unambiguous. Any typographical or grammatical errors should be corrected at revision, so please note any specific errors here.

Reviewer #1: Yes

Reviewer #2: Yes

Reviewer #4: Yes

**********

6. Review Comments to the Author

Please use the space provided to explain your answers to the questions above. You may also include additional comments for the author, including concerns about dual publication, research ethics, or publication ethics. (Please upload your review as an attachment if it exceeds 20,000 characters)

Reviewer #1: I want to congratulate the authors on a much improved manuscript and acknowledge how thoroughly and seriously they addressed the comments received in the prior round of review. I have only very minor comments to offer at this stage, the most substantial of the bunch refer to the methods section and should be very quick to resolve.

INTRO

--The abbreviation "ED" is not defined at first use

--The authors write "5x" rather than "five times"

--Typically, numerals less than 10 are spelled out (i.e. it should be "six months" rather than "6 months"). I will defer to the journal's style guide on this one

--Typically, U.S. is only used as an adjective (i.e. U.S. residents) and United States is only used as a noun (i.e. in the United States)

--There appears to be an extra carriage return on line 92

METHODS

--The mention of sampling limitations in the first paragraph is good, but the authors should further clarify how this is likely affecting the results. Right now, that impact on results is insinuated but not stated explicitly.

--It does not appear as though the authors took steps to control for for multiple comparison in their chi-squared analyses (listed in Table 2). This may be de facto handled by the fact that the table only indicates when p<0.001, but at least some mention of the authors awareness of the risk of false discovery and source of confidence in their presented data should be made. Even as a footnote to the table would be fine.

RESULTS

at least one of the "p"s in table 2 is capitalized.

Reviewer #2: (No Response)

Reviewer #4: The authors have thoroughly and adequately responded to the the reviewers comments. I ave no additional comments.

**********

7. PLOS authors have the option to publish the peer review history of their article (what does this mean?). If published, this will include your full peer review and any attached files.

If you choose “no”, your identity will remain anonymous but your review may still be made public.

**Do you want your identity to be public for this peer review?**   For information about this choice, including consent withdrawal, please see our Privacy Policy.

Reviewer #1: No

Reviewer #2: No

Reviewer #4: No

**********

[NOTE: If reviewer comments were submitted as an attachment file, they will be attached to this email and accessible via the submission site. Please log into your account, locate the manuscript record, and check for the action link "View Attachments". If this link does not appear, there are no attachment files.]

To ensure your figures meet our technical requirements, please review our figure guidelines: https://journals.plos.org/plosone/s/figures

You may also use PLOS’s free figure tool, NAAS, to help you prepare publication quality figures: https://journals.plos.org/plosone/s/figures#loc-tools-for-figure-preparation.

NAAS will assess whether your figures meet our technical requirements by comparing each figure against our figure specifications.

# Author comment: Author response to Decision Letter 2

**DOI:** 10.1371/journal.pone.0351233.r004

5 May 2026

May 5, 2026

PLOS One

Ref: PONE-D-25-43064

Title: Prevalence of Criminal Legal Involvement Among Emergency Department Patients: Insights from the National Survey on Drug Use and Health 2021-2023

Authors: Vidya Eswaran, MD, Jun-Hong Chen PhD, Utsha G. Khatri, MD, Phillip L Marotta, PhD, Michael G Vaughn, PhD

Andrea K. Knittel, MD, PhD

Academic Editor, PLOS One Editorial Office

Dear Dr. Knittel,

We appreciate the insightful comments and suggestions provided by you and the review team for our manuscript, “Prevalence of Criminal Legal-Involvement Among Emergency Department Patients: Insights from the 2023 National Survey on Drug Use and Health”. Our team has carefully reviewed these insights and has revised our work accordingly. We also have highlighted relevant changes in the manuscript.

Reviewer #1: I want to congratulate the authors on a much improved manuscript and acknowledge how thoroughly and seriously they addressed the comments received in the prior round of review. I have only very minor comments to offer at this stage, the most substantial of the bunch refer to the methods section and should be very quick to resolve.

Response: Thank you for your comments and for your close review of the updated manuscript. We have addressed your comments as noted below.

Introduction

Comment 1: The abbreviation "ED" is not defined at first use

Response: This has been corrected on line 69.

Comment 2: The authors write "5x" rather than "five times"

Response: This has been corrected on line 73.

Comment 3: Typically, numerals less than 10 are spelled out (i.e. it should be "six months" rather than "6 months"). I will defer to the journal's style guide on this one

Response: This has been corrected throughout the manuscript.

Comment 4: Typically, U.S. is only used as an adjective (i.e. U.S. residents) and United States is only used as a noun (i.e. in the United States)

Response: This has been corrected throughout the manuscript.

Comment 5: There appears to be an extra carriage return on line 92

Response: This has been corrected.

METHODS

Comment 6: The mention of sampling limitations in the first paragraph is good, but the authors should further clarify how this is likely affecting the results. Right now, that impact on results is insinuated but not stated explicitly.

Response: Thank you for this comment. We have added the following to the methods section, line 161: “It is thus possible that our results may understate the prevalence of CLI in this population.”

Comment 7: It does not appear as though the authors took steps to control for multiple comparison in their chi-squared analyses (listed in Table 2). This may be de facto handled by the fact that the table only indicates when p<0.001, but at least some mention of the authors awareness of the risk of false discovery and source of confidence in their presented data should be made. Even as a footnote to the table would be fine.

Response: Thank you for this comment. We have completed a simple chi square test applied with a Bonferroni correction as well as complex sample weighting, and found the same significance patterns (i.e., significance is still significance).

We have added the following footnote to the bottom of Table 2: “To address potential false discovery, a Bonferroni correction was applied, and statistical significance was assessed using the adjusted significance threshold. The results remained unchanged.

RESULTS

Comment 8: at least one of the "p"s in table 2 is capitalized.

Response: This has been corrected in Table 2.

Thank you for taking the time to review and suggest improvements to this manuscript. We look forward to your feedback and appreciate opportunities to continue to revise as needed.

Sincerely,

Vidya Eswaran, MD MAS

Washington University School of Medicine

660 South Euclid Avenue, Box 8072,St. Louis, Missouri 63110

Phone: 314-362-4126 | vidya@wustl.edu

Attachment Submitted filename: NSDUH ED Arrest PLOSOne Response to Reviewers2.docx

# Editor report: Decision Letter 2

**DOI:** 10.1371/journal.pone.0351233.r005

**Authors:** Andrea K. Knittel

26 May 2026

Prevalence of Criminal Legal Involvement Among Emergency Department Patients: Insights from the National Survey on Drug Use and Health 2021-2023

PONE-D-25-43064R2

Dear Dr. Eswaran,

We’re pleased to inform you that your manuscript has been judged scientifically suitable for publication and will be formally accepted for publication once it meets all outstanding technical requirements.

Within one week, you’ll receive an e-mail detailing the required amendments. When these have been addressed, you’ll receive a formal acceptance letter and your manuscript will be scheduled for publication.

An invoice will be generated when your article is formally accepted. Please note, if your institution has a publishing partnership with PLOS and your article meets the relevant criteria, all or part of your publication costs will be covered. Please make sure your user information is up-to-date by logging into Editorial Manager at Editorial Manager® and clicking the ‘Update My Information' link at the top of the page. For questions related to billing, please contact billing support.

If your institution or institutions have a press office, please notify them about your upcoming paper to help maximize its impact. If they’ll be preparing press materials, please inform our press team as soon as possible -- no later than 48 hours after receiving the formal acceptance. Your manuscript will remain under strict press embargo until 2 pm Eastern Time on the date of publication. For more information, please contact onepress@plos.org.

Kind regards,

Andrea K. Knittel

Academic Editor

PLOS One

Additional Editor Comments (optional):

Reviewers' comments:

# Editor report: Acceptance letter

**DOI:** 10.1371/journal.pone.0351233.r006

**Authors:** Andrea K. Knittel

PONE-D-25-43064R2

PLOS One

Dear Dr. Eswaran,

I'm pleased to inform you that your manuscript has been deemed suitable for publication in PLOS One. Congratulations! Your manuscript is now being handed over to our production team.

At this stage, our production department will prepare your paper for publication. This includes ensuring the following:

* All references, tables, and figures are properly cited

* All relevant supporting information is included in the manuscript submission,

* There are no issues that prevent the paper from being properly typeset

You will receive further instructions from the production team, including instructions on how to review your proof when it is ready. Please keep in mind that we are working through a large volume of accepted articles, so please give us a few days to review your paper and let you know the next and final steps.

Lastly, if your institution or institutions have a press office, please let them know about your upcoming paper now to help maximize its impact. If they'll be preparing press materials, please inform our press team within the next 48 hours. Your manuscript will remain under strict press embargo until 2 pm Eastern Time on the date of publication. For more information, please contact onepress@plos.org.

You will receive an invoice from PLOS for your publication fee after your manuscript has reached the completed accept phase. If you receive an email requesting payment before acceptance or for any other service, this may be a phishing scheme. Learn how to identify phishing emails and protect your accounts at https://explore.plos.org/phishing.

If we can help with anything else, please email us at customercare@plos.org.

Thank you for submitting your work to PLOS ONE and supporting open access.

Kind regards,

PLOS ONE Editorial Office Staff

on behalf of

Dr. Andrea K. Knittel

Academic Editor

PLOS One
